# Artificial cell membrane binding thrombin constructs drive in situ fibrin hydrogel formation

**DOI:** 10.1038/s41467-019-09763-0

**Published:** 2019-04-23

**Authors:** Robert C. Deller, Thomas Richardson, Rebecca Richardson, Laura Bevan, Ioannis Zampetakis, Fabrizio Scarpa, Adam W. Perriman

**Affiliations:** 1 School of Cellular and Molecular Medicine, University of Bristol, Bristol, BS8 1TD UK; 2 Bristol Centre for Functional Nanomaterials, University of Bristol, Bristol, BS8 1FD UK; 3 School of Physiology, Pharmacology and Neuroscience, University of Bristol, Bristol, BS8 1TD UK; 4 Bristol Composites Institute (ACCIS), University of Bristol, Bristol, BS8 1TR UK; 5 Present Address: School of Engineering, University of Liverpool, Liverpool, L69 3GH UK

**Keywords:** Cell delivery, Regenerative medicine, Mesenchymal stem cells, Biomaterials - cells, Gels and hydrogels

## Abstract

Cell membrane re-engineering is emerging as a powerful tool for the development of next generation cell therapies, as it allows the user to augment therapeutic cells to provide additional functionalities, such as homing, adhesion or hypoxia resistance. To date, however, there are few examples where the plasma membrane is re-engineered to display active enzymes that promote extracellular matrix protein assembly. Here, we report on a self-contained matrix-forming system where the membrane of human mesenchymal stem cells is modified to display a novel thrombin construct, giving rise to spontaneous fibrin hydrogel nucleation and growth at near human plasma concentrations of fibrinogen. The cell membrane modification process is realised through the synthesis of a membrane-binding supercationic thrombin-polymer surfactant complex. Significantly, the resulting robust cellular fibrin hydrogel constructs can be differentiated down osteogenic and adipogenic lineages, giving rise to self-supporting monoliths that exhibit Young’s moduli that reflect their respective extracellular matrix compositions.

## Abstract (web summary)

The incorporation of cells into tissue engineering scaffolds can be a major challenge. Here, the authors report on anchoring thrombin to cell membranes for the in situ formation of fibrin scaffolds around the modified cells, demonstrate scaffold formation in vitro and show cell survival in vivo.

## Introduction

As advanced cellular-based therapies approach clinical translation, there is an increasing demand for new cell specific matrices that provide improved therapeutic performance^1^. However, rational matrix design is extremely challenging, as cell phenotype and fate are reliant on a wide range of scaffold-dependent environmental factors, which include cell adhesion, chemical composition, cell receptor stimulation, micro- and nanomorphology, and mechanical stiffness^2,3^. These factors come into play almost immediately, as in vitro tissue engineering typically begins with the seeding and adhesion of cells onto a biocompatible and biodegradable scaffold, which acts as a surrogate for the extracellular matrix (ECM)^4^. As the cells proliferate and/or differentiate, they produce ECM that gradually replaces the scaffold material, giving rise to a structurally self-supporting entity^5^.

A range of biocompatible natural polymers have been used to produce transient hydrogel scaffolds for tissue engineering, including chitosan, gelatin, collagen, hyaluronic acid and fibrin^6^. Of these biopolymer-based scaffolds, fibrin hydrogels are amongst the most popular, as they can be readily produced in situ at room temperature via proteolytic cleavage^7^. Fibrin formation in vivo occurs as a response to injury, culminating with the proteolytic cleavage of prothrombin to thrombin, which is a serine protease that catalyses the polymerization of fibrinogen to fibrin, resulting in the formation of a fibrin hydrogel clot^8^. Importantly, fibrin hydrogels display specific peptide motifs, such as RGD, which promote adhesion via integrin signalling, and mediate the recruitment, activation and presentation of growth factors (e.g. BMP-2 and TGF-β1), which help regulate osteogenic and chondrogenic differentiation in human mesenchymal stem cells (hMSCs)^9–11^. Moreover, a key advantage of (fibrin) hydrogels over solid porous scaffolds such as poly(lactide-co-glycolide) is their ability to be delivered via injection^12^, although extrusion-based shear stress can result in a reduction in cell viability^13^.

Cell membrane engineering is emerging as a premier approach for cell augmentation, as it can be used to rapidly provide additional cell functionality, including immunoevasion, adhesion and homing^14^. However, there are few examples where cell membrane modifications have been used to drive in situ scaffold formation. Wu et al.^15^ and Cruise et al.^16^ irradiated cells coated in the photosensitive dyes eosin and polyethylene (glycol) diacrylate to generate hydrogel structures on the surface of lung adenocarcinoma cells and porcine islets, respectively, although both systems required synthetic monomers and an external source of radiation.

**Fig. 1:**
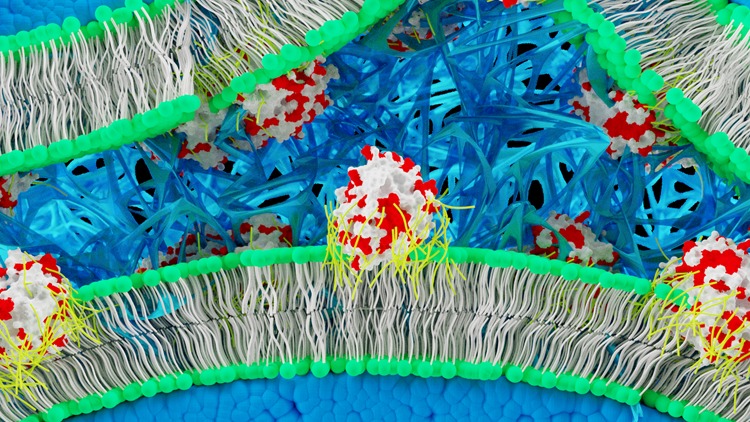
Schematic showing in situ fibrin hydrogel formation from the membranes of bone-marrow derived human mesenchymal stem cells (hMSCs). Artificial membrane binding thrombin constructs comprising supercationic thrombin molecules (white) surrounded by a polymer surfactant corona (yellow) that associates with surface exposed cationic (red) residues spontaneously insert into bilayer regions of hMSC plasma membranes. In the presence of fibrinogen, the membrane-immobilised thrombin catalyses fibrin formation (blue fibres) within the interstitial spaces between the cells giving rise to a self-supporting hydrogel monolith

Here, we describe a methodology involving the synthesis of supercationic thrombin-polymer surfactant complexes that spontaneously bind the plasma membrane of hMSCs and drive in situ fibrin hydrogel nucleation and growth (Fig. 1). The resulting self-supporting hydrogel constructs support high levels of metabolic activity, and provide a matrix for adipogenic and osteogenic differentiation. Moreover, we demonstrate that this cell functionalisation methodology is facile and can be used to inject thrombin labelled GFP-expressing fibroblasts into an in vivo zebrafish skin wound model.

## Results

### Artificial membrane binding thrombin synthesis

**Fig. 2:**
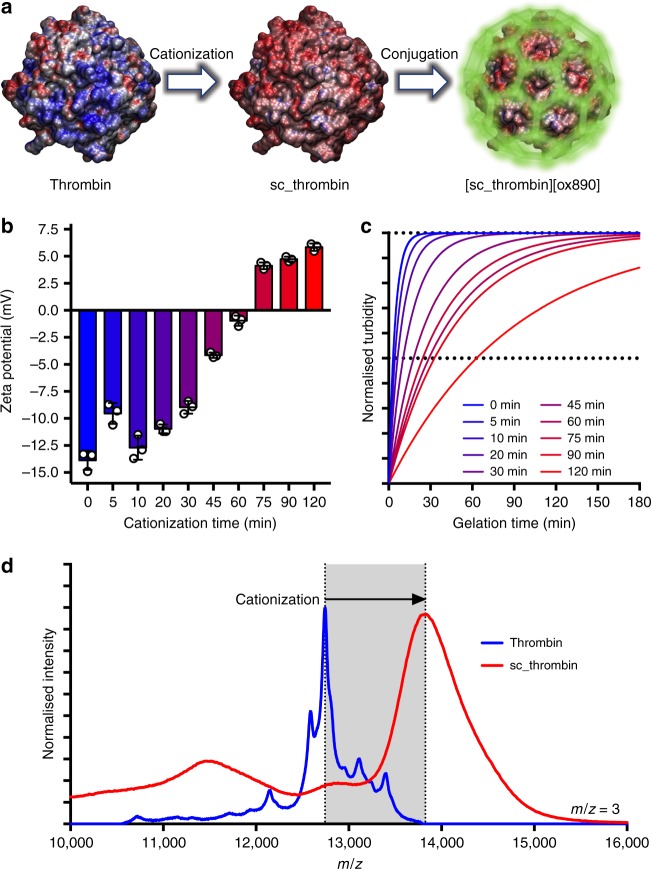
The synthesis and characterisation of the supercationic thrombin-polymer surfactant conjugate. **a** Schematic showing the electrostatic surface potential of native and supercationic thrombin (sc_thrombin) (PDB; *1UVS*) at pH 7, highlighting the anionic (blue) and cationic (red) charged regions. Generation of the polymer surfactant corona (green halo) via electrostatic coupling of glycolic acid ethoxylate 4-nonylphenyl ether (ox890) to sc_thrombin gives [sc_thrombin][ox890]. **b** Zeta potential (ca. pH7; *n* = 3) of thrombin as a function of cationization times (0–120 min). Data reported as means ± standard deviation (s.d.). **c** Rate of fibrinogen solution (3.125 mg mL^−1^) gelation as measured by changes in turbidity (600 nm) catalysed by sc_thrombin subjected to various cationization times (0–120 min). Data shown as one-phase association curves of raw data. **d** MALDI-TOF MS spectra (*m*/*z* = 3) of native and sc_thrombin (60 min). Source data are provided as a Source Data file

The synthesis of the cell membrane binding thrombin (bovine) complex was performed using a two-step process (Fig. 2a). The initial step involved the kinetically controlled cationization of thrombin via N-(3-dimethylaminopropyl)-N′ethylcarbodiimide hydrochloride (EDC) mediated covalent attachment of cationic N,N′-dimethyl-1,3-propanediamine (DMPA) to surface exposed carboxylic acid functional groups to generate an active supercationic thrombin (sc_thrombin) construct (Supplementary Fig. 1)^17^. Thrombin not only contains glutamic acid (21) and aspartic acid (18) residues, but also acid functional groups arising from posttranslational modifications, including up to 10 ɣ-carboxyglutamyl residues that each contain two carboxylic acid groups^18^ and carboxylic acid groups resulting from the N-linked glycosylation of three asparagine residues^19^. Accordingly, the reaction conditions were carefully controlled (pH, temperature, and the number of EDC and DMPA equivalents) and the enzyme cationization reaction progress was monitored using zeta potentiometry, which shifted from −13.9 ± 0.9 to +5.8 ± 0.3 mV over a 2 h period (Fig. 2b). As the active site of thrombin contains the serine protease catalytic triad including a reactive aspartic acid (Asp189)^20^, it was necessary to concomitantly evaluate the activity of the thrombin during the reaction. This was achieved by monitoring the increase in fibrinogen solution turbidity resulting from fibrin gel formation, which showed a steady decrease in reaction rate as the cationization reaction progressed over the 2 h period (Fig. 2c). To balance the effective cationic surface charge density required for electrostatic surfactant conjugation with high enough levels of enzymatic activity, the reaction was quenched at 60 min and these conditions used for all subsequent experiments. Matrix assisted laser desorption ionisation time of flight mass spectroscopy performed on the resulting sc_thrombin (Fig. 2d), showed an average increase in mass of 3.25 kDa, which signified charge reversal (negative to positive) at 39 sites.

While it has been shown that supercationic proteins such as supercharged green fluorescent protein have a high membrane binding affinity, strong electrostatic interactions with the anionic sulfonated proteoglycans of the glycocalyx results in rapid endocytosis^21,22^. This transient behaviour has been used to good effect for the delivery of siRNA^23^, as has the cationic polymer poly(ethylenimine) (PEI) for nucleic acid^24^ and protein^25^ delivery. However, our principle objective was to display the active thrombin at the cell surface in order to nucleate a fibrin gel from the plasma membrane. Therefore, a second step involved electrostatically conjugating the anionic polymer surfactant glycolic acid ethoxylate 4-nonylphenyl ether (ox890), which comprises an anionic carboxylic acid headgroup, a hydrophilic poly(ethylene)glycol central moiety (E40) and a hydrophobic nonylphenyl tail (Supplementary Fig. 2 & Supplementary Methods). Significantly, we have recently shown that this approach can be used to generate a polymer surfactant corona on the surface of myoglobin that reconfigures to insert into the plasma membrane of hMSCs^17^. Here, electrostatic conjugation of the polymer surfactant (≈120 molecules per protein) to the sc_thrombin to yield [sc_thrombin][ox890] resulted in an negative shift in the zeta potential distribution (Supplementary Fig. 3). Dynamic light scattering experiments (Supplementary Fig. 4) performed on solutions of thrombin, sc_thrombin and [sc_thrombin][ox890], gave particle size number distributions centred at approximately 5 nm, with a small increase in hydrodynamic diameter distribution after surfactant conjugation, which is consistent with previous reports^17,26–28^.

Surfactant conjugation did not result in a reduction in catalytic activity (cf. sc_thrombin) (Supplementary Fig. 5), and confocal fluorescence microscopy from fibrinogen solutions supplemented with Alexa Fluor^®^ 594 dye-labelled fibrinogen showed the presence of a complex fibrin network after treatment with either thrombin, sc_thrombin or [sc_thrombin][ox890] (Supplementary Fig. 6 & Supplementary Methods). Moreover, unconfined compression testing of the resulting self-supporting structures gave Young’s moduli (Supplementary Fig. 7) that were consistent with soft fibrin hydrogels^29^.

### Thrombin construct membrane affinity and hydrogel formation

**Fig. 3:**
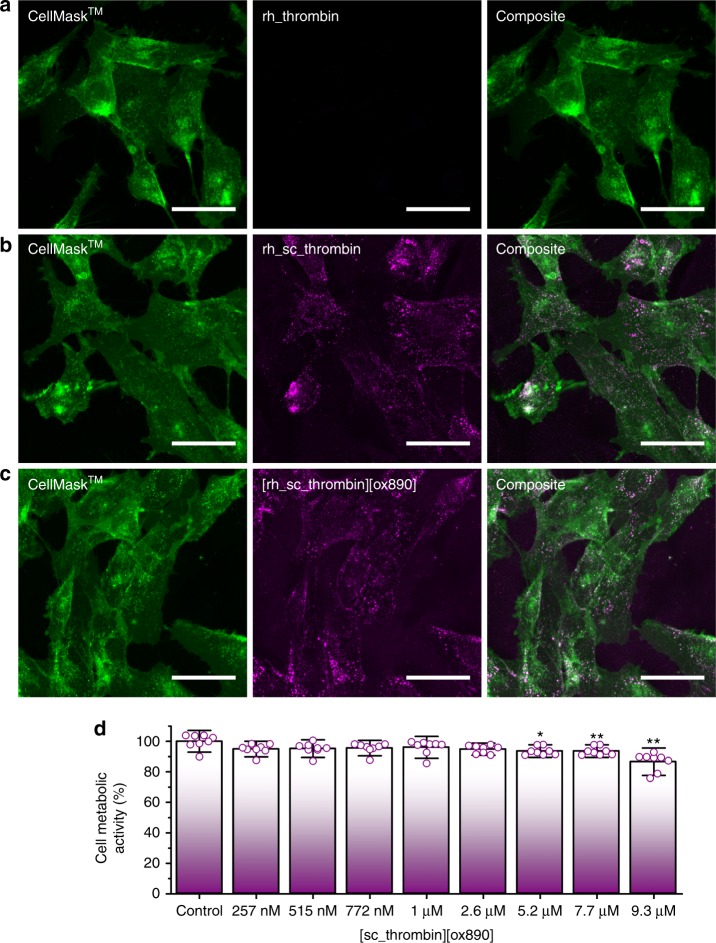
Evaluation of rh_thrombin, rh_sc_thrombin and [rh_sc_thrombin][ox890] hMSC plasma membrane affinity. Cells labelled with CellMask™ Deep Red (green) and corresponding rhodamine labelled thrombin (magenta). **a** Native (rh_thrombin) thrombin at *T* = 0 h. **b** Cationised (sc_rh_thrombin) thrombin at *T* = 0 h. **c** Polymer surfactant conjugate ([rh_sc_thrombin][ox890]) thrombin at *T* = 0 h. Scale bars represent 50 µm. **d** MTS cell metabolic activity assay (*n* = 8) of hMSCs after labelling with [sc_thrombin][ox890] compared to unlabelled controls. Data reported as means ± s.d. Dunnett’s test; *p ≤ 0.05, **p ≤ 0.001. Source data are provided as a Source Data file

A clinical application for thrombin functionalised cells could be readily derived from in vitro tissue engineered constructs that utilise transient fibrin scaffolds. Accordingly, bone marrow derived hMSCs with well-characterised differentiation pathways (e.g. adipogenic, chondrogenic and osteogenic) were selected to assess membrane adhesion and cell metabolic activity^30^. Initially, a monolayer of hMSCs was incubated with 1 μM Rhodamine B labelled analogues (rh_thrombin, rh_sc_thrombin and [rh_sc_thrombin][ox890]) for 20 min and subsequently washed to remove any unbound protein. Significantly, this resulted in increases in Rhodamine B fluorescence from hMSCs labelled with either rh_sc_thrombin or [rh_sc_thrombin][ox890], signifying insertion and retention to the plasma membrane of the hMSCs. Thereafter, hMSCs were labelled with a plasma membrane specific dye (CellMask™ DeepRed) and imaged immediately (Fig. 3a–c & Supplementary Fig. 8), which showed colocalisation with the rh_sc_thrombin or [rh_sc_thrombin][ox890] constructs, which confirmed plasma membrane binding. Furthermore, the presence of the rh_sc_thrombin and [rh_sc_thrombin][ox890] construct could be detected over a period of at least 6 h (Supplementary Movie 1), although a significant proportion of both rh_sc_thrombin and [rh_sc_thrombin][ox890] was endocytosed during this period. To test in situ fibrin formation from the [rh_sc_thrombin][ox890] functionalised cells over time, a 10 mg mL^−1^ fibrinogen solution supplemented with Alexa Fluor® 488 dye-labelled fibrinogen was added to a confluent layer of the hMSCs. Here, time-lapse confocal fluorescence microscopy shows nucleation and growth from the cells in both the *x*–*y* plane and *z* direction (Supplementary Movie 2 & Supplementary Fig. 9).

MTS assays^31^ were performed to measure the relative metabolic activity of hMSCs labelled with the [sc_thrombin][ox890], which showed no significant reduction up to concentrations as high as 2.6 μM (*p* = 0.087) (Fig. 3d). Accordingly, a 1 μM [sc_thrombin][ox890] incubation concentration was selected for all subsequent experiments, as this afforded suitable catalytic activity, membrane loading potential and biocompatibility, and was within the physiological concentration range of (pro)thrombin in plasma^32^. Moreover, control cell metabolic activity assays were also performed using the neat oxidised surfactant (ox890) and its synthetic precursor (Supplementary Fig. 10 & Supplementary Methods), which showed no significant cytotoxicity up to the highest concentrations tested (25 mM).

**Fig. 4:**
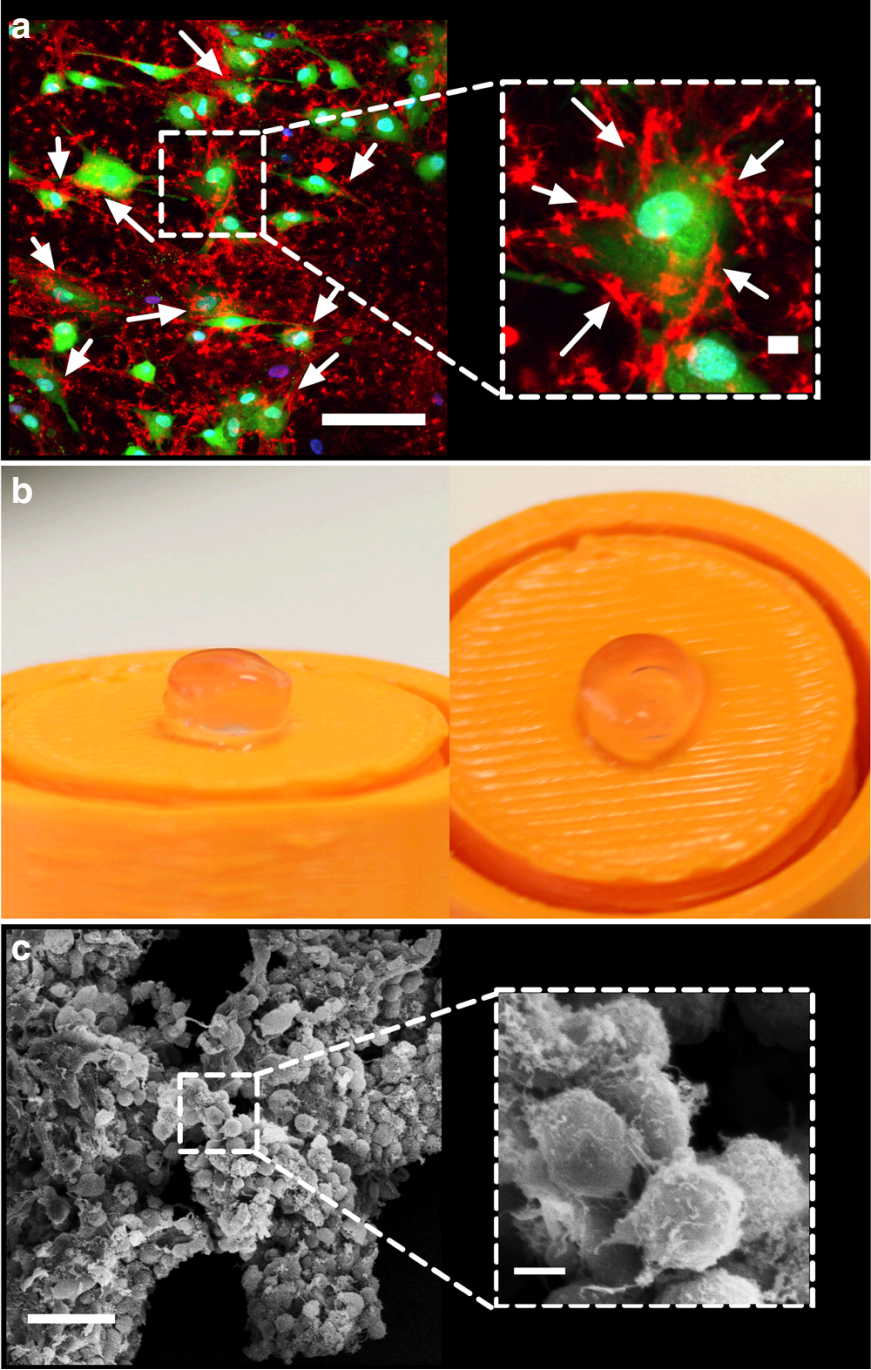
Evaluation of cellular fibrin hydrogel formation. **a** Live-cell confocal fluorescence micrograph (2D) showing Alexa Fluor® 594 labelled fibrin (red) nucleation after 20 min from the surface of hMSCs stained with Calcein AM (green) and Hoechst 33342 (blue). Insert shows single cell at higher magnification. Scale bars represent 100 and 10 μm (insert). **b** Photographs of the free-standing fibrin hydrogels 24 h after gelation formed via [sc_thrombin][ox890] hMSCs labelling. **c** Scanning electron micrograph of [sc_thrombin][ox890] modified hMSCs aggregates (3D) after 24 h showing a surface catalysed fibrin matrix. Insert shows cells at higher magnification. Scale bars represent 50 and 5 μm (insert)

In order to further assay the enzymatic activity of the hMSC membrane-bound [sc_thrombin][ox890] construct, a low (1 mg mL^−1^) fibrinogen solution supplemented with Alexa Fluor^®^ 594 dye labelled fibrinogen was introduced in complete medium without FBS (to prevent FBS-driven fibrin formation). Significantly, confocal fluorescence microscopy images show fibrin structures emanating from the plasma membrane of the hMSC monolayer (Fig. 4a & Supplementary Movie 3) after 20 min, along with limited fibrin networks in acellular regions, indicating gel nucleation from tissue culture treated plastic bound [sc_thrombin][ox890]. This is in contrast to hMSCs devoid of [sc_thrombin][ox890] labelling and in the absence of FBS where no fibrin formation was observed after 60 min (Supplementary Fig. 11). The membrane labelling protocol was then modified to generate 3D fibrin hydrogel constructs containing 1 × 10^7^ cell mL^−1^ (Dia. = 6.5 mm; Vol. = 100 μL; ≈1 × 10^6^ cells) (Fig. 4b). Here, confluent hMSCs were labelled with 1 μM [sc_thrombin][ox890], re-suspended in FBS-free culture media supplemented with 6 mg mL^−1^ fibrinogen, and pipetted into wells coated in a 1 wt.% agarose gel. Fibrin gelation was allowed to proceed for a period of 60 min prior to the addition of an equal volume of high glucose media supplemented with 20% (v/v) FBS and 0.1 TIU mL^−1^ aprotinin to limit fibrin degradation^33^. After 24 h, the constructs were fixed and imaged using scanning electron microscopy (Fig. 4c), which showed dense cellular aggregates with individual cells surrounded by a 3D fibrin matrix. Z-stacks produced using live cell confocal fluorescence microscopy showed a well-dispersed distribution of hMSCs, uniformly suspended within the fibrin hydrogel matrix (Supplementary Movie 4 & Supplementary Fig. 12).

### hMSC differentiation and mechanical properties

**Fig. 5:**
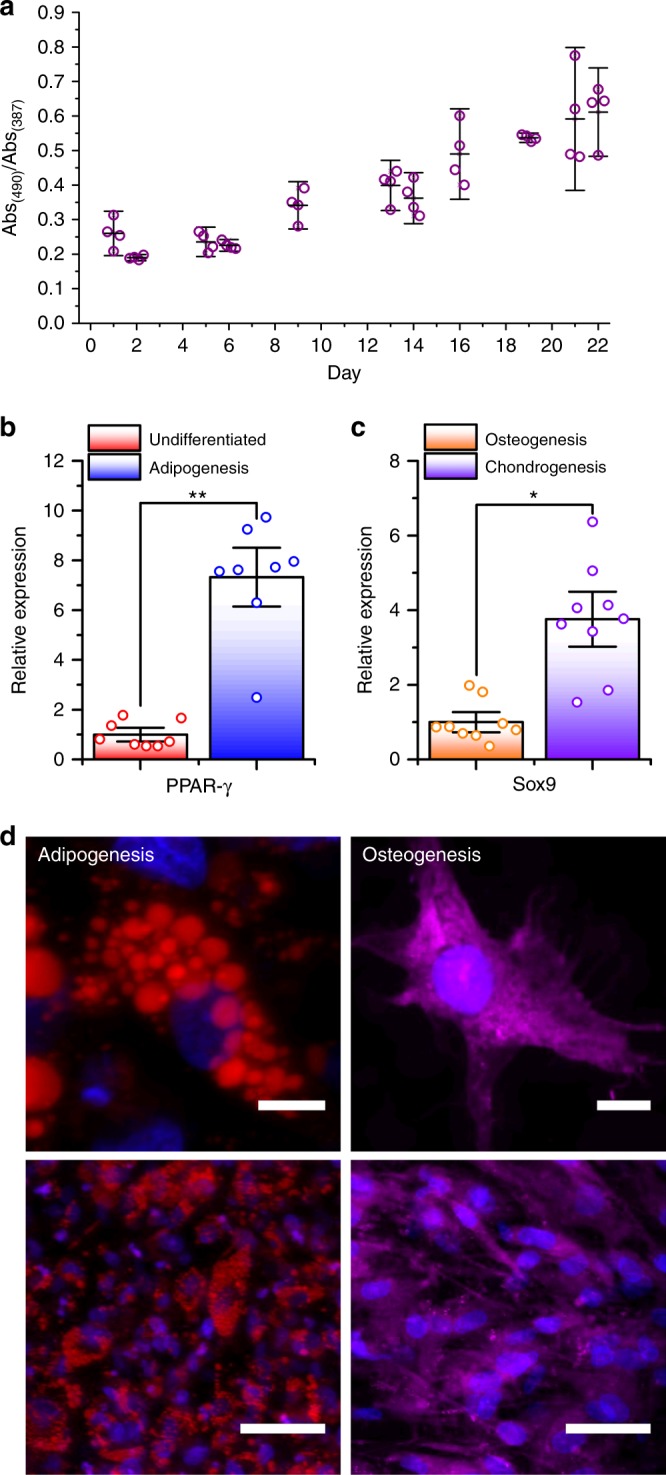
Cell metabolic activity and differentiation potential of hMSCs within in situ formed fibrin hydrogel constructs. **a** MTS cell metabolic activity assay (*n* ≥ 4) of hMSCs at various intervals after fibrin construct formation. Data reported as the mean ratio of product:reactant ± s.d. **b** Relative expression (*n* = 8) of *PPAR-γ* in [sc_thrombin][ox890] coated hMSCs within catalysed fibrin, cultured in standard or adipogenic medium for 14 days. Data reported as means ± standard error of the mean (s.e.m.). One-tailed paired *t*-test; **p ≤ 0.01. **c** Relative expression (*n* = 9) of *SOX9* in [sc_thrombin][ox890] coated hMSCs within catalysed fibrin, cultured in chondrogenic or osteogenic medium for 7 days. Data reported as means ± s.e.m. One-tailed paired *t*-test; *p ≤ 0.05. **d** Confocal fluorescence micrographs (Z-projections) of individual (top) and groups of (bottom) cells within fibrin constructs with scale bars representing 10 and 50 μm, respectively. Cells stained with Hoechst 33342 (blue) differentiated down adipogenic (left) and stained with oil red o (red) and osteogenic (right) and stained with alizarin red (magenta) lineages. Source data are provided as a Source Data file

In order to investigate the ability of the fibrin hydrogel system to sustain 3D cultures for the extended periods of time required for tissue engineering, the metabolic activity of hMSCs within the fibrin constructs was monitored over 22 days using a MTS cell metabolic assay. Cells were cultured in a medium that maintained hMSC multipotency to avoid changes in innate cell metabolic activity due to differentiation, which allowed the relative increase in cell number to be estimated. Significantly, a constant increase in the ratio of the formazan product (490 nm) to the MTS reactant (387 nm) was observed over the entire period, which indicated sustained levels of hMSC proliferation (Fig. 5a).

Reverse transcription quantitative polymerase chain reaction (RT-qPCR) experiments were performed to monitor the early-stages of differentiation of the [sc_thrombin][ox890] modified hMSCs in the fibrin hydrogel system. The *PPAR-γ* gene is a pivotal ligand-activated transcription factor that upon activation is upregulated and drives hMSCs towards an adipogenic fate^34^. Accordingly, upregulated *PPAR-γ* expression was used as an early indicator of adipogenic differentiation (14 days), which showed a 7-fold increase when the hMSCs were cultured in adipogenic media (cf. standard media) (Fig. 5b). To probe the capability of cells to undergo chondrogenesis, the relative expression of the chondrogenic gene *SOX9* was explored^35^. *SOX9* is upregulated in response to the addition of chondrogenic factors (e.g. TGF-β3) and downregulated in the presence of osteogenic factors (e.g. BMP-2), with its expression linked to the activity of the osteoresponsive gene *RUNX2*^36^. Here, *SOX9* expression in the fibrin constructs supplemented with chondrogenic media resulted in a 4-fold increase in *SOX9* expression (cf. osteogenic media) after 7 days (Fig. 5c). However, no significant increase in *RUNX2* expression was apparent in the fibrin constructs supplemented with osteogenic media (cf. standard media) after 7 days (Supplementary Fig. 13).

Following on from the RT-qPCR experiments, the hMSC fibrin constructs were differentiated down adipogenic or osteogenic lineages over a 21 day period to enable the potential for development of typical phenotypic traits^30,37^. Aside from visual changes in cell morphology, analysis of the resulting constructs were probed by the addition of specific fluorescent stains relevant for each lineage. This included Oil Red O for lipid vacuole formation during adipogenesis^38^ and Alizarin Red for calcium deposition resulting from osteogenesis^39^. For the modified cells exposed to the adipogenic media, confocal fluorescence microscopy images showed clusters of lipid vacuoles, emanating from cells with a globular morphology, which was consistent with the formation of mature adipocytes (Fig. 5d). Conversely, modified cells exposed to the osteogenic media exhibited extensive calcium deposition, signifying osteogenesis, which was accompanied by subtle changes away from a spindle-like morphology (but not cuboidal), highlight the ongoing transition toward the formation of fully mature osteoblasts (Fig. 5d)^40^. Both phenotypes were also observed across a wider population of cells, liberated, re-plated (overnight) and imaged in 2D (Supplementary Fig. 14).

**Fig. 6:**
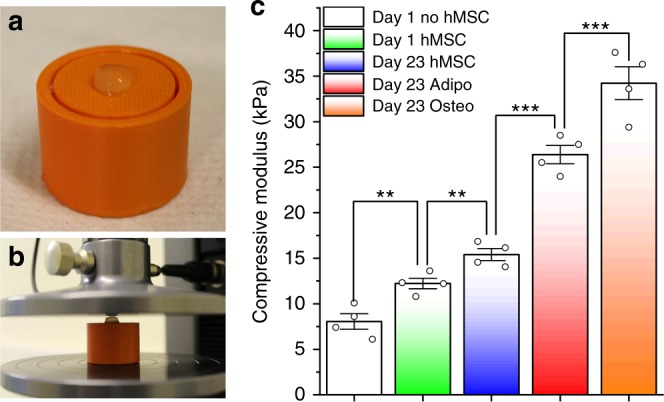
Compression testing of 3D hMSC fibrin hydrogel constructs. Images of a 3D hMSC fibrin hydrogel construct (Dia. = 8.0 mm; depth ∼4.2 mm; ≈4 × 10^6^ cells) before (**a**) and during (**b**) unconfined compression testing. **c** Compressive modulus (*n* = 4) of fibrin gels containing [sc_thrombin][ox890] coated hMSCs cultured for 23 days in undifferentiated, adipogenic or osteogenic media, [sc_thrombin][ox890] coated hMSCs cultured for 1 day in undifferentiated media and native thrombin (200 nM) catalysed (no hMSCs) after 1 day. Data reported as the means ± s.e.m. Tukey’s test; **p ≤ 0.05 ***p ≤ 0.005. Source data are provided as a Source Data file

The differentiation pathways of hMSCs are dependent on the mechanical properties of their environment, with stiffer interfaces generally favouring an osteogenic fate and softer surfaces adipogenesis^41–43^. In practice, this means that ECM formation during tissue engineering can provide positive feedback to differentiation, as it can directly impact on the stiffness of the surrounding environment. For example, the deposition of calcium phosphate during osteogenic biomineralization can result in an increase in stiffness in the surrounding environment, which in turn can stabilise the osteoblast phenotype^44^. To explore the impact of ECM formation on the mechanical properties, unconfined compression testing (Fig. 6a, b) was performed on the constructs (Dia. = 8.0 mm; Vol. = 400 µL; ≈ 4 × 10^6^ cells) formed from 6 mg mL^−1^ fibrinogen solutions cultured in either osteogenic, adipogenic or standard expansion medium for a period of 23 days. These were compared to constructs cultured for 1 day (containing hMSCs) in standard expansion media, and with constructs devoid of hMSCs and catalysed using 200 nM native thrombin. Significant differences between each system were observed, with day 1 Young’s compressive moduli of 8.1 kPa in fibrin only constructs, which increased to 12.2 kPa in the presence of hMSCs. After 23 days, hMSCs undergoing osteogenesis exhibited a much higher stiffness (34.2 kPa), when compared with those undergoing adipogenesis (24.6 kPa) or multipotency culture conditions (15.4 kPa) (Fig. 6c). These results indicate that the cell membrane nucleated fibrin structures are amenable to scaffold-matrix remodelling by the differentiated cells, and that the resulting ECM influences the bulk compressive mechanical properties^45^.

### In vivo localisation of thrombin-modified fibroblasts

**Fig. 7:**
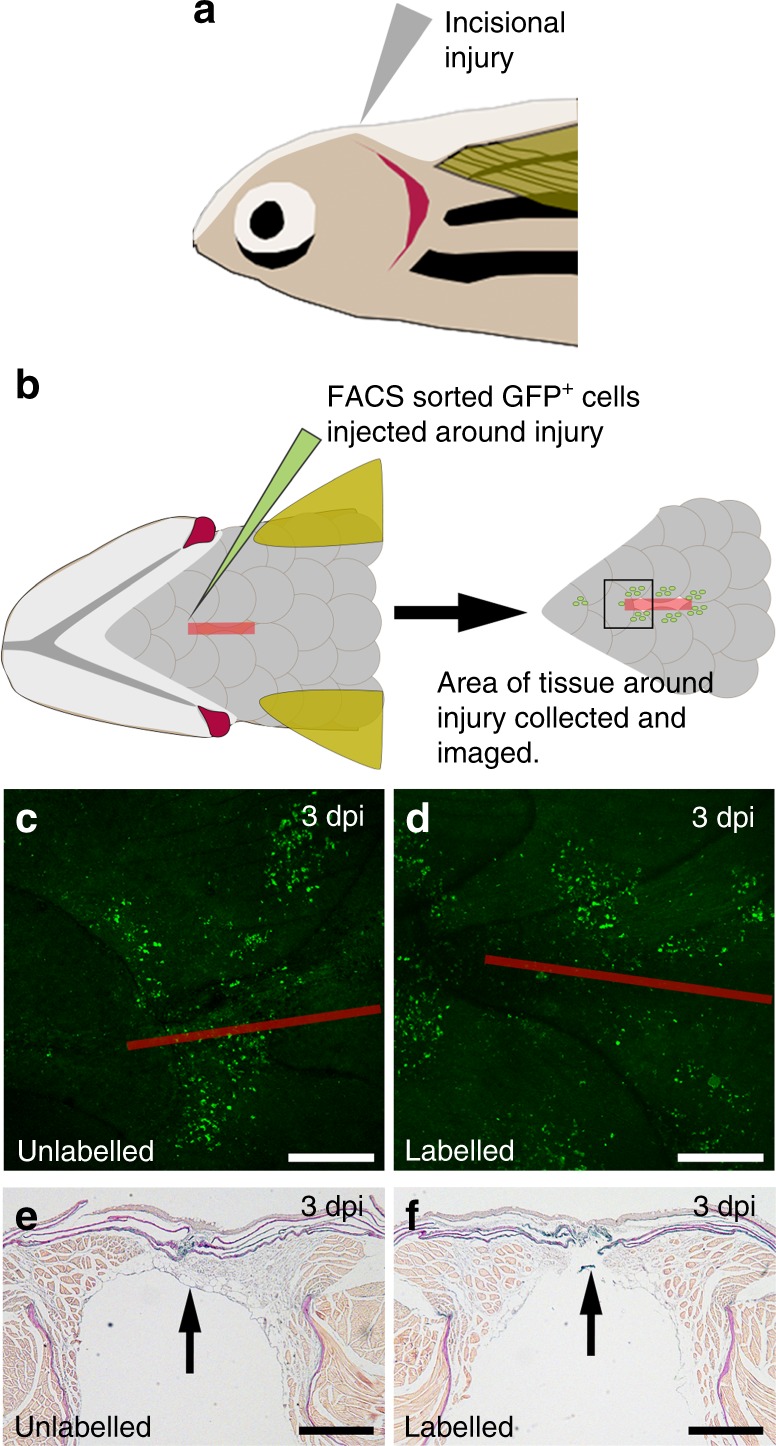
In vivo zebrafish injury and [sc_thrombin][ox890] labelled GFP + fibroblast addition. Schematic representation of the in vivo adult zebrafish injury model. **a** Wildtype (non-transgenic) recipient zebrafish were anaesthetized and a 4 mm incisional injury made on the ventral upper thorax. A lateral view is shown. **b** Unlabelled or [sc_thrombin][ox890] labelled, FACS sorted GFP+ fibroblasts were injected at six sites around the edge of the incisional injury. At the desired time-point, fish were sacrificed and the tissue surrounding the incision was fixed, imaged and embedded for sectioning. A ventral view is shown. Ventral view of the area of tissue surrounding the incision at 3 dpi following transfer of **c** unlabelled or **d** [sc_thrombin][ox890] labelled GFP+ fibroblasts. Similar numbers of cells were retained at all wounds. The red line depicts the approximate position of the incisional injury which is fully re-epithelialized at this stage. Sections through the injury region at 3 dpi following transfer of **e** unlabelled or **f** [sc_thrombin][ox890] labelled GFP+ fibroblasts. No obvious differences were observed between wounds containing labelled or unlabelled cells. Arrows indicate the position of the incision. All scale bars represent 250 µm

Although the development of the membrane re-engineering approach described above could prove advantageous for in vitro tissue engineering, the ability to produce thrombin coated cells ad hoc that could be injected into a site of injury provides new opportunities for in vivo tissue engineering. Here, an injury site would be preferable as endogenous fibrinogen is freely available within intravascular fluids such as blood plasma at between 1.5 and 4 mg mL^−1^ ^46^. However the clinical use of exogenous fibrinogen within fibrin sealants may also permit administration to other sites^47^. Accordingly, we performed preliminary cell transplant studies using an in vivo zebrafish model system to examine the isolation, labelling, delivery and survival of a different cell type, namely, primary zebrafish fibroblasts. Zebrafish (*Danio rerio*) are an established model organism and provide an excellent platform for live cell imaging with numerous cell type specific fluorophore-expressing strains available (Supplementary Fig. 15)^48^. Zebrafish have also been considered as a model organism for studying thrombolytic and haemostatic processes^49^ and furthermore, haemostatic activity has been observed by the infusion of activated bovine thrombin to zebrafish in vivo as a method to assess the availability of clottable fibrinogen in antithrombin III mutants^50^. Consequently, fibroblasts from GFP-expressing *ET37* fish^51^ (Supplementary Fig. 16) were isolated by FACS (Supplementary Fig. 17) prior to immediate labelling with the [sc_thrombin][ox890] conjugate. Cells were then washed and re-suspended, with donor labelled (GFP+) fibroblasts (micro)injected around the site of a fresh ventral incisional injury in adult, non-transgenic recipient zebrafish (Fig. 7a, b). Significantly, similar numbers of viable unlabelled and thrombin-functionalised labelled cells were observed at 3 days post (micro)injection (dpi), suggesting normal cell survival (Fig. 7c, d). Macroscopic observations and histological analysis of sections through the incisional injury revealed no adverse effects between fish injected with labelled and unlabelled fibroblasts (Fig. 7e, f), however further investigations will be required to determine precise effects on specific wound healing processes.

Herein, we described the synthesis and characterisation of a new membrane active thrombin construct that can be used to drive in situ fibrin formation from the plasma membranes of stem cells. The resulting hydrogel constructs support high levels of viability and proliferation in hMSCs cultures over 22 days, which can be readily differentiated to produce self-supporting tissue engineered constructs. Unconfined compression testing showed that differentiation of the hMSCs down well-defined adipogenic and osteogenic pathways directly impacted the Young’s moduli of the resulting structures, which indicates high levels of integration between the nucleated fibrin hydrogel and the resulting ECM.

The facile membrane labelling strategy and tissue engineering protocols described above provide a new approach to both in vitro and in vivo tissue engineering, although a detailed understanding of the temporal behaviour of the membrane-bound thrombin constructs (e.g., internalisation and deactivation of the enzyme) would need to be ascertained before clinical translation. This is key, as controlling the concentration of bioavailable thrombin, is a fundamental requirement in the development of haemostatic products.

## Methods

### Materials

All materials unless otherwise specified were purchased from Sigma-Aldrich Company Ltd (Poole, UK) or ThermoFisher Scientific Ltd (Loughborough, UK) and were of the highest available purity.

### Human mesenchymal stem cells

Human mesenchymal stem cells (hMSCs) derived from human bone marrow were sourced with informed consent from healthy donors from Southmead Hospital, Bristol, UK in full accordance with Bristol Southmead Hospital Research Ethics Committee guidelines (reference #078/01), North Bristol NHS Trust.

### Cationization, surfactant oxidation and conjugation

For protein cationization a 2 mg mL^**−**1^ solution of protein (Bovine Thrombin, Sigma-Aldrich Company Ltd, Cat. No. T7326 (https://www.uniprot.org/uniprot/P00735)) was dissolved in 60 mM HEPES (pH7). A solution (adjusted to pH 7) of DMPA equivalent to approximately 150-fold the number of cationizable sites was added along with solid N-(3-dimethylaminopropyl)-N′ethylcarbodiimide hydrochloride (EDC) equivalent to ~34-fold the number of cationizable sites, yielding a final protein concentration of 1 mg mL^**−**1^. The complete mixture was then adjusted to pH 6.5 and stirred for 60 min (or as indicated) at 25 °C. Afterwards, excess DMPA and EDC were removed by diluting 4-fold with 20 mM HEPES (pH7) at 4 °C and using 10 K MWCO spin concentrators. Dilution and spin concentration as aforementioned was repeated a minimum of five times to limit further cationization and protein concentration verified using a Pierce™ BCA protein assay kit (ThermoFisher Scientific Ltd, Cat. No. 23225). Samples were then stored at −20 °C prior to further use for up to 72 h. Surfactant oxidation was achieved by the complete oxidation of the terminal hydroxyl group of IGEPAL Co890 to yield a terminal carboxylic acid functional group. Two grams of IGEPAL Co890 dissolved in 50 mL of deionized water was mixed with 30 mg 2,2,6,6,-tetramethyl-1-piperidinyloxyl (TEMPO), 50 mg sodium bromide and 5 mL of a sodium hypochlorite solution containing 10-15 % available chlorine. The solution was adjusted to pH 11 and then stirred overnight at room temperature before the reaction was quenched with the addition of 10 mL of ethanol and adjusted to pH 1 prior to a solvent extraction with three 80 mL aliquots of chloroform. The combined chloroform fractions were washed with three 80 mL aliquots of ddH_2_O at pH 1 and chloroform removed by rotary evaporation (40 °C/150 mbar). The resulting product was dissolved in 40 mL of ethanol at 65 °C and then recrystallized overnight at −20 °C. The following day the ethanol was decanted with this process repeated once more prior to removing any residual ethanol by rotary evaporation (50 °C/120 mbar) to yield a fully oxidized product. Surfactant conjugation was achieved by dissolving the oxidized product directly into the desired protein solution at room temperature at a concentration equivalent to 1.4 the total number of glutamic acid, aspartic acid, lysine and arginine residues and stirred for a period 1 h and protein concentration verified using a Pierce™ BCA protein assay kit. Samples were then used immediately or stored at −20 °C prior to further use for up to 72 h.

### Zeta potentiometry

Zeta potentiometry was performed on a Zetasizer Nano ZSP (Malvern Instruments Ltd, UK) with a minimum of three replicates taken at the indicated pH values and a temperature of 25 °C. The values reported show the mean (mV) with error bars representing ± standard deviation (s.d.).

### Matrix assisted laser desorption ionisation time of flight mass spectrometry

Matrix assisted laser desorption ionisation time of flight (MALDI-TOF) mass spectrometry was performed on a UltrafleXtreme mass spectrometer (Bruker (UK) Ltd, Coventry, UK) using a matrix of containing a saturated solution of α-Cyano-4-hydroxycinnamic acid in acetonitrile with 0.1% trifluoroacetic acid in a 1:1 ratio of sample in H_2_O mixed and spotted onto a 384-well plate with a ground steel surface prior to ionization and detection (100% laser power/+ve ion mode).

### rh_thrombin, rh_sc_thrombin and [rh_sc_thrombin][ox890] co-localisation

Preparations of 1 μM rh_thrombin, rh_sc_thrombin and [rh_sc_thrombin][ox890] were produced by the addition of a 15-fold molar excess of NHS-rhodamine (DMSO) to Thrombin (PBS) at 4 °C for 120 min. Excess NHS-rhodamine was removed using a spin concentrator (MWCO: 10 kDa) with 20 mM HEPES (pH 7) buffer. Conjugation efficiency was then determined using UV-visible spectroscopy by measuring relative intensities at 280 nm (protein) and 555 nm (rhodamine)^52^. Cells were labelled with 1 μM of the desired thrombin solutions in DMEM media (no FBS) with 60 mM HEPES (pH7) for 20 min prior to washing with PBS. Thereafter, cells were labelled with CellMask™ DeepRed following the as supplied instructions prior to imaging in complete medium ((DMEM low (1000 mg L^**−**1^) glucose supplemented with 100 μg mL^**−**1^ streptomycin, 2 mM Glutamax, 100 U mL^−1^ penicillin, 10% (v/v) FBS, 5 ng mL^**−**1^ fibroblast growth factor).

### sc_thrombin catalytic activity

Assessment of sc_thrombin (0.05 mg mL^**−**1^) catalytic activity subjected to various cationization times (0–120 min) was completed by measuring changes in turbidity (600 nm) at 25 °C by UV-visible spectroscopy (Agilent Technologies LDA U.K. Limited, Stockport, U.K.) of a 3.125 mg mL^**−**1^ solution of fibrinogen (total volume; 200 μL) with increasing in turbidity indicative of fibrin formation.

### [sc_thrombin][ox890] Cytotoxicity

Sterile (0.22 μm filtered) stock solutions of [sc_thrombin][ox890] at 10.30 μM (≈0.38 mg mL^−1^) in 20 mM HEPES pH7 were prepared and diluted to the appropriate protein concentration with a final buffer concentration of 60 mM HEPES buffer (pH7). To start, complete medium was aspirated from confluent low passage (<5) hMSCs in a 96 well plate (1 × 10^4^ cells/well) and cells then rinsed with 100 μL of PBS prior to the addition of 100 μL of the appropriate solution (0-9.27 μM/eight replicates) to cells and incubated for a period of 20 min at 37 °C/5% CO_2_. After 20 min cells were rinsed twice with 100 μL PBS prior to assessment of cell metabolic activity by the MTS cell metabolic assay. MTS cell metabolic activity assays were performed using a CellTiter 96® AQ_ueous_ One Solution Cell Proliferation Assay kit (Promega UK Ltd, Cat. No. G3582) following the as supplied instructions with hMSCs incubated for a period of 3 h prior to analysis by UV-visible spectroscopy at 490 nm with values expressed as a percentage in comparison to untreated controls.

### Fibrin construct formation

Confluent hMSCs (<4) were cultured in 175 cm^2^ flasks in complete medium. Up to 32 wells (as desired) within a 96-well plate were precoated with 50 μL 1  wt.% agarose, with surrounding wells filled with 200 μL of water containing 100 μg mL^**−**1^ streptomycin, 100 U mL^−1^ penicillin and 250 μg mL^**−**1^ amphotericin B. Afterwards a sterile 7.5 mg mL^**−**1^ stock of human fibrinogen (Sigma-Aldrich Company Ltd, Cat. No. F4883) in complete medium (without FBS) was prepared at 37 °C in preparation for fibrin formation. In order to label confluent hMSCs, cells were washed with 25 mL PBS and labelled with 25 mL (37 °C) of 1 μM [sc_thrombin][ox890] in 60 mM HEPES (pH7) subsequently added prior to incubation at 37 °C/5% CO_2_ for 20 min. Afterwards cells were immediately washed with 25 mL PBS twice and trypsinised (8 mL) for 5 min in order to liberate adherent hMSCs into a suspension, to which 16 mL of complete medium is added (in order to inhibit further trypsin activity). The resulting suspension was then centrifuged (400 × *g*, 25 °C, 5 min) with the supernatant discarded and replaced with 25 mL of complete medium (without FBS) and centrifuged (400 × *g*, 25 °C, 5 min) once more with the supernatant discarded, this washing (i.e. FBS removal) step was then repeated once more. The visible cell pellet was then resuspended in complete medium (without FBS) to a total volume between 75–150 μL (dependant on the number of constructs to be created). A 20 μL aliquot of resuspended hMSCs (5 × 10^7^ cells mL^**−**1^ was then added to 80 μL 7.5 mg mL^**−**1^ human fibrinogen preplaced into a precoated (agarose) well and gently mixed by pipetting. The resulting mixture was then incubated at 37 °C/5% CO_2_ on an orbital rotator (50 rpm) for 60 min to promote and consolidate fibrin gel formation (6 mg mL^**−**1^). Afterwards cells were supplemented with 100 μL of the relevant medium (e.g. adipogenic) containing 0.1 TIU mL^**−**1^ aprotinin with 3–5 media changes per week for up to 23 days.

### Scanning electron microscopy

Scanning electron microscopy (SEM) was performed on a JSM IT300 Scanning Electron Microscope (Jeol Ltd., Japan). Fibrin constructs were prepared as aforementioned and after 24 h fixed in 4% (v/v) Paraformaldehyde for 60 min. Samples were then dehydrated to 100% (v/v) ethanol by 2 min immersions in 70, 80 and 90% (v/v) ethanol. Samples were then critically point dried in a Leica EM CPD300 (Leica Microsystems Ltd., UK) mounted onto SEM stubs and coated in gold using a high resolution Emitech K550X sputter coater (Quorum Technologies Ltd., UK) prior to imaging.

### Fibrin construct imaging

All live hMSC confocal microscopy was performed on a Leica SP8 AOBS confocal laser scanning microscope attached to a Leica DM I6000 inverted epifluorescence microscope (Leica Microsystems (UK) Ltd, UK) in a temperature controlled (37 °C) environment. Prior to imaging, cells (labelled or unlabelled) where indicated were stained with Calcein AM (1 μM) and Hoechst 33342 (5 μg μL^**−**1^) for a period of 20 min prior to imaging using the appropriate excitation and emission wavelengths. Fibrinogen stocks were produced ad hoc containing 0.1 mg of Alexa Fluor^®^ 594 labelled human fibrinogen (ThermoFisher Scientific Ltd. Cat. No. F13193) per 0.9 mg of human fibrinogen (unless otherwise stated) freshly prepared in complete medium minus FBS which upon [sc_thrombin][ox890] mediated conversion to fibrin allows for visualisation of the fibrin network using the appropriate excitation (594 nm) and emission (600+ nm) wavelengths.

### Quantitative reverse transcriptase PCR

Fibrin constructs (hMSCs derived from three patients each with three biological replicates) were prepared as aforementioned and cultured with the appropriate differentiation media for a period of up to 14 days (as required) and isolated by trypsin digestion to yield a suspension of cells. RNA extraction was performed using a RNeasy® Micro kit (Qiagen Ltd., UK. Cat. No 74004) and followed the as supplied instructions. RNA concentration was then determined using a P-330 nanophotometer (Implen GmbH, Germany) prior to cDNA synthesis using an Omniscript® reverse transcriptase kit (Qiagen Ltd., UK. Cat. No. 205111) following the as supplied instructions and 50 ng of template RNA. Quantitative reverse transcriptase PCR (RT-qPCR) (with two technical duplicates) was then performed on a StepOne Plus RT-qPCR instrument (Applied Biosystems Inc., USA) with TaqMan® Universal Master Mix (II) (ThermoFisher Scientific Ltd. Cat. No. 4440043) and corresponding TaqMan® gene expression assays for *SOX9* (UniGene ID; Hs.647409), *PPAR-γ* (UniGene ID; Hs.162646), *RUNX2* (UniGene ID; Hs.535845) and (housekeeping) *GAPDH* (UniGene ID; Hs.544577). The raw C_t_ values were collated and the Δ*C*_t_ values determined and exponentiated to give the relative expression levels reported.

### Live cell mechanical testing

All mechanical testing was performed on a STARRET FMS-500-L2 Force Measurement System (The L.S. Starrett Company Limited, UK) fitted with a 100 N load cells at room temperature. Constructs constituting a volume of 400 μL fibrin cast within a 48-well plate (Dia. = 11.0 mm; depth ∼4.2 mm;) were created using the same methodology as aforementioned, with all components including cell number scaled up proportionally (i.e. 4-fold) with a final concentration of fibrin of 6 mg mL^**−**1^. All samples were incubated at 37 °C/5% CO_2_ prior to analysis. Samples with hMSCs were supplemented with 400 μL of the appropriate medium (i.e. complete, adipogenic, osteogenic) and aprotinin (0.1 TIU/mL). For the 23 day constructs, media was subsequently changed at frequent (five times per week) intervals prior to analysis to permit differentiation and the development of phenotypic characteristics. Constructs devoid of hMSCs were catalysed by thrombin at a final concentration of 200 nM. Prior to analysis, all media was removed and the samples gently blotted to remove any residual liquid. Samples were then cut using an 8 mm (Dia.) biopsy punch to create uniform fibrin discs. Each sample was then mounted onto a PLA stage and subjected to destructive compression testing at a rate of 1 mm min^**−**1^. The compressive modulus (kPa) of each sample was then calculated with the values reported representing the mean (*n* = 4) ± standard error of the mean (s.e.m.).

### Zebrafish cell labelling

Wildtype and *ET37* Zebrafish (*Danio rerio*) were bred and kept in accordance with approval from the local ethical review committee at the University of Bristol and in accordance with UK Home Office regulations. All fish used were aged 4–18 months. *ET37* fish express GFP under the control of an unknown enhancer previously shown to label larval mesenchymal cells. To obtain fibroblasts for labelling and adoptive transfer, adult *ET37* fish were anaesthetised in 0.13% MS-222 (Sigma-Aldrich Company Ltd, Cat. No. A5040) and the caudal fin resected. Pooled samples of fins from four fish were collected into PBS containing 10 mM HEPES, 30 mM taurine and 5.5 mM glucose and cells dissociated by the addition of 0.25% trypsin, 12.5 M CaCl_2_ and 5 mg ml^**−**1^ Collagenase II (Worthington Biochemical Corp.; Cat. No. LS004176). Dissociated cells were suspended in Zebrafish medium which comprised, Leibovitz medium (L-15) containing 0.3 mM glutamine, 0.8 mM CaCl_2_, 50 μg mL^**−**1^ Streptomycin, 50 U mL^−1^ Penicillin and 2% (v/v) FBS. *ET37*+ fibroblasts were sorted for GFP expression on a BD Biosciences InFlux high-speed Fluorescence activated cell sorter (BD Biosciences INC., San Jose, California). Sorted GFP+ cells were labelled with 1 μM [sc_thrombin][ox890] as a suspension of 1 × 10^5^ cells mL^**−**1^ for 20 min incubated at 30 °C/5% CO_2_ in Zebrafish medium plus 100 μg mL^**−**1^ Normocin (InvivoGen Inc.; Cat. No. ant-nr-1) with FBS at 10% (v/v). Cells were then centrifuged (250 × *g*, 4 °C, 10 min) with the resulting supernatant discarded. Cells were then resuspended (1 × 10^6^ cells mL^**−**1^) and washed once more by centrifugation before resuspension at a final concentration of 4 × 10^7^ cells mL^**−**1^ prior to microinjection.

### Zebrafish adoptive cell transfer

Wildtype, non-fluorescent recipient fish were anaesthetised in 0.13% MS-222 and placed ventral side up in a pre-cut sponge soaked in aquarium water containing anaesthetic. A 4-mm incision was made through the skin and the pericardial sac directly above the heart. This area was chosen as the underlying dermis is thicker than other sites and the procedure is routinely preformed. Approximately 1000 GFP+ labelled or unlabelled cells in a 50 nL volume were microinjected at six sites around the edge of the incisional injury and the fish allowed to recover in fresh system water without anaesthetic.

### Zebrafish imaging and staining procedures

At the desired time-points, recipient fish were terminated and the area around the incision dissected and fixed in 4% (v/v) paraformaldehyde (PFA) for 1 h at room temperature, washed twice in PBS and mounted in 1.5 wt.% low melting point agarose. Images of endogenous GFP expression were acquired on a Leica DM6000 SP8 AOBS upright confocal laser scanning microscope. For histological analysis, skin samples were decalcified in 0.5 M EDTA (pH 7.4) at room temperature for 5 days, embedded in paraffin wax, sectioned and stained as described previously^53^. Sections of 8 μm thickness were stained with AFOG using standard protocols.

### Data analysis

Data interpretation, modelling, numerical analysis and statistical analysis were performed using Microsoft Excel for Mac 2011 (Microsoft Corporation, WA, U.S.A), Prism 7 for Mac OS X (Graph Pad Inc., CA, U.S.A.) and OriginPro 2015 (64-bit) Sr2 (OriginLab Corp., MA, USA). Statistical tests and confidence intervals detailing significant differences are as described in the figure legends of the appropriate figure.

### Reporting summary

Further information on research design is available in the Nature Research Reporting Summary linked to this article.

## Supplementary information

Supplementary Information

Description of Additional Supplementary Files

Supplementary Movie 1

Supplementary Movie 2

Supplementary Movie 3

Supplementary Movie 4

Reporting Summary

Source Data

## Data Availability

All data supporting the research findings in this study are available from the corresponding author upon reasonable request. The source data underlying Figs. 2b, 3d, 5a, 5b, 5c, 6c and Supplementary Figs. 7a, 10 and 13 are provided as a Source Data file.
