# Supplementary material for: Artificial cell membrane binding thrombin constructs drive in situ fibrin hydrogel formation
Source: Nat Commun. 2019 Apr 23;10:1887. doi: 10.1038/s41467-019-09763-0 (PMC6478844; doi:10.1038/s41467-019-09763-0)
Supplement: Supplementary file 1 — Supplementary Information [file 41467_2019_9763_MOESM1_ESM.pdf]

Supplementary Information

Artificial cell membrane binding thrombin constructs drive *in situ* fibrin hydrogel formation

Deller *et. al.*

Supplementary Figures

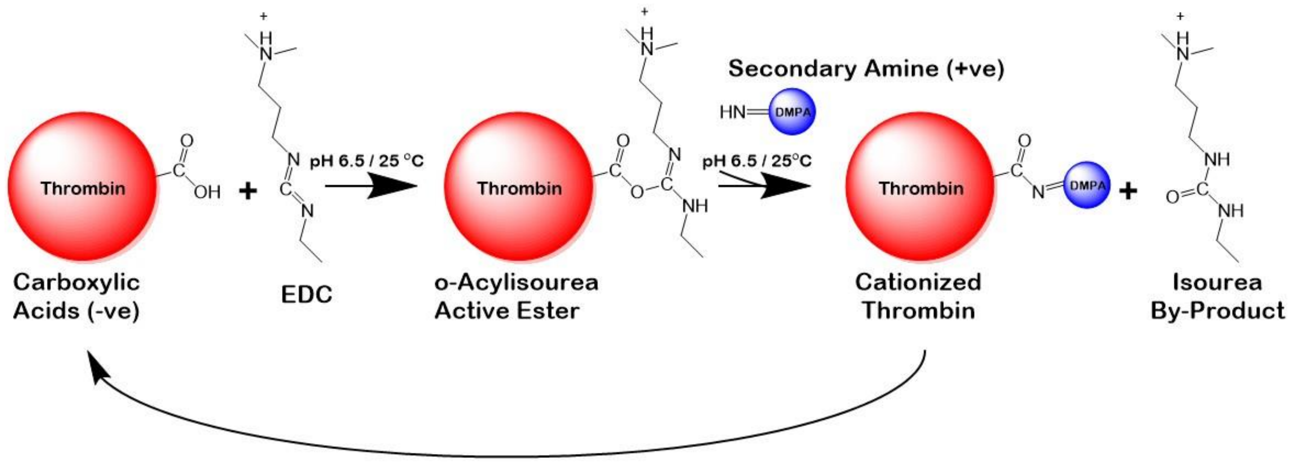

Supplementary Figure 1 | Schematic of protein cationization via the N-(3-dimethylaminopropyl)-N’ethylcarbodiimide hydrochloride (EDC) mediated covalent attachment of cationic N,N’-dimethyl-1,3-propanediamine (DMPA) to carboxylic acid functional groups generating a supercationic construct.

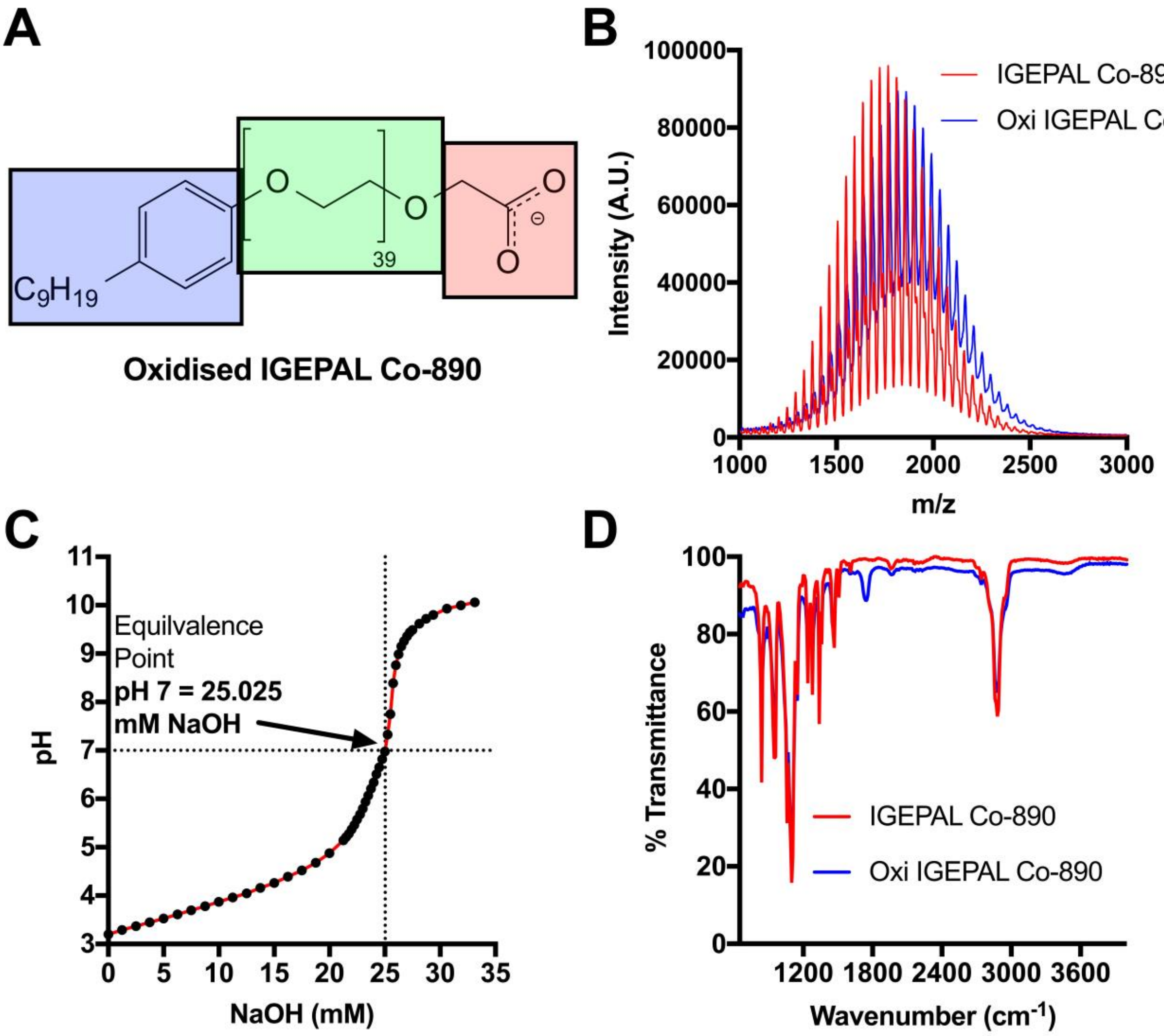

Supplementary Figure 2 | Characterisation of oxidised IGEPAL Co-890 surfactant. **A.** Structure of oxidised IGEPAL Co-890, highlighting the modified anionic headgroup (red), poly(ethylene)glycol linker (green) and hydrophobic nonylphenyl membrane binding domain (blue). **B.** MALDI-TOF mass spectroscopy of IGEPAL Co-890 and oxidised IGEPAL Co-890 highlighting an increase in mass and no significant changes in polydispersity. **C.** Acid-Base titration of oxidised IGEPAL Co-890 highlighting near 100% conversion of the terminal hydroxyl group to the desired carboxylic acid. **D.** FT-IR of IGEPAL Co-890 and oxidised IGEPAL Co-890 highlighting the presence of a carbonyl (C=O stretch; 1740 cm^{-1}) group indicative of a carboxylic acid.

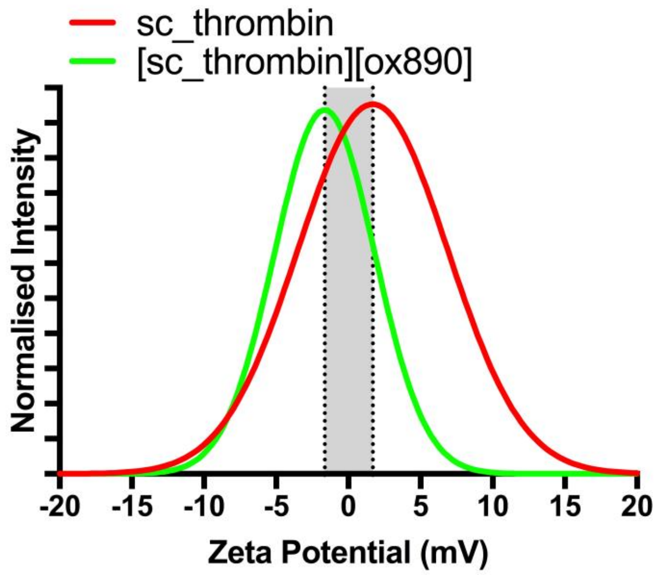

Supplementary Figure 3 | Zeta Potentiometry distribution of sc_thrombin (+1.7 mV) and [sc_thrombin][ox890] (-1.6 mV) at pH7 in 20 mM HEPES. Data shown as normalised Gaussian distributions of raw data.

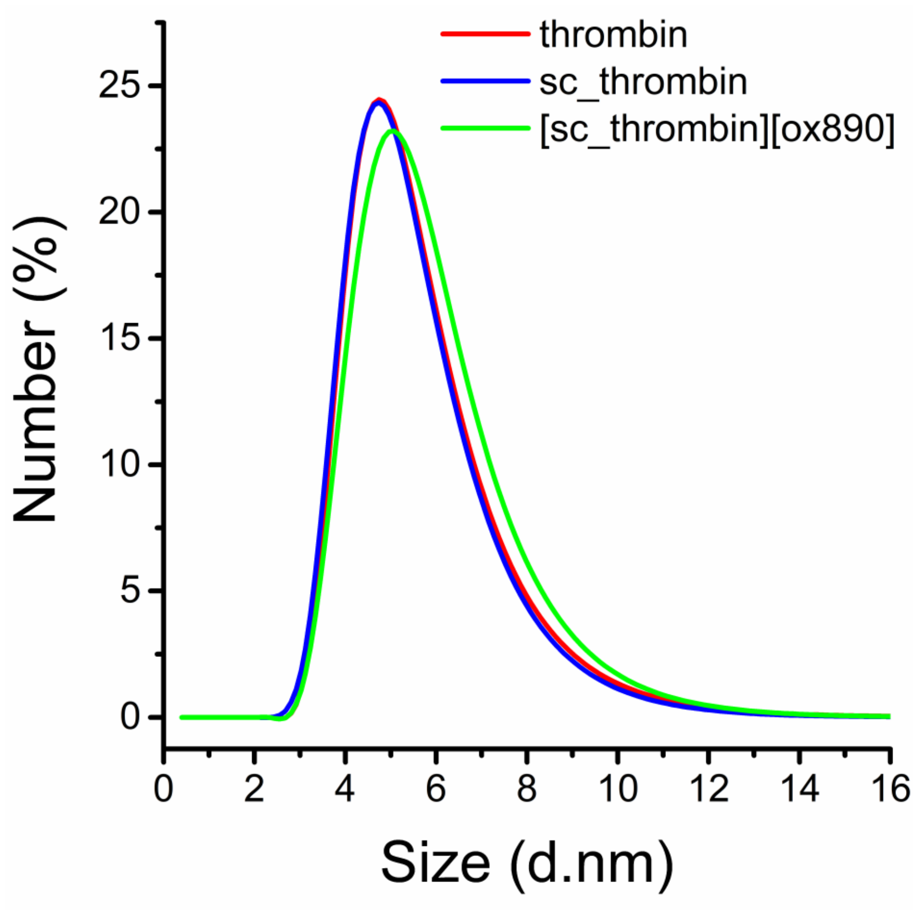

Supplementary Figure 4 | Dynamic light scattering measurements of thrombin preparations. Native Thrombin (red), sc_thrombin (blue) and [sc_thrombin][ox890] (green). All measurements were attained in 20 mM HEPES (pH6.5) at 25 °C. Curves represent % values of number distribution data.

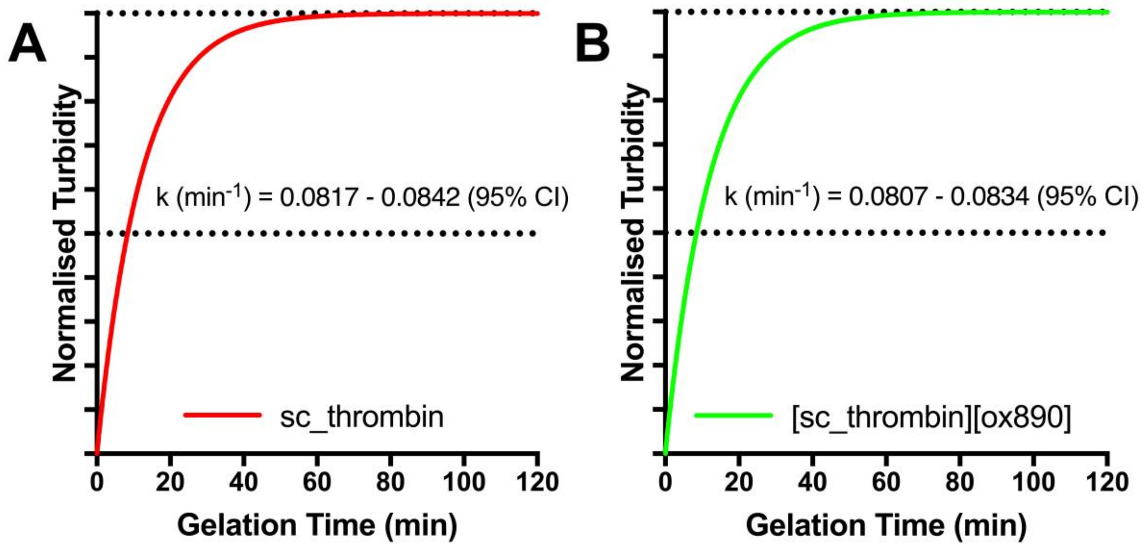

Supplementary Figure 5 | Rate of fibrin (2.5 mg.mL^{-1}) gelation as measured by changes in turbidity (600 nm) catalysed by **A.** sc_thrombin and **B.** [sc_thrombin][ox890]. Data shown as one-phase association curves with Y=0 and Plateau =100 of the raw data (>700 data points) with corresponding rate constants (k; min^{-1}) listed with a 95% confidence interval (CI).

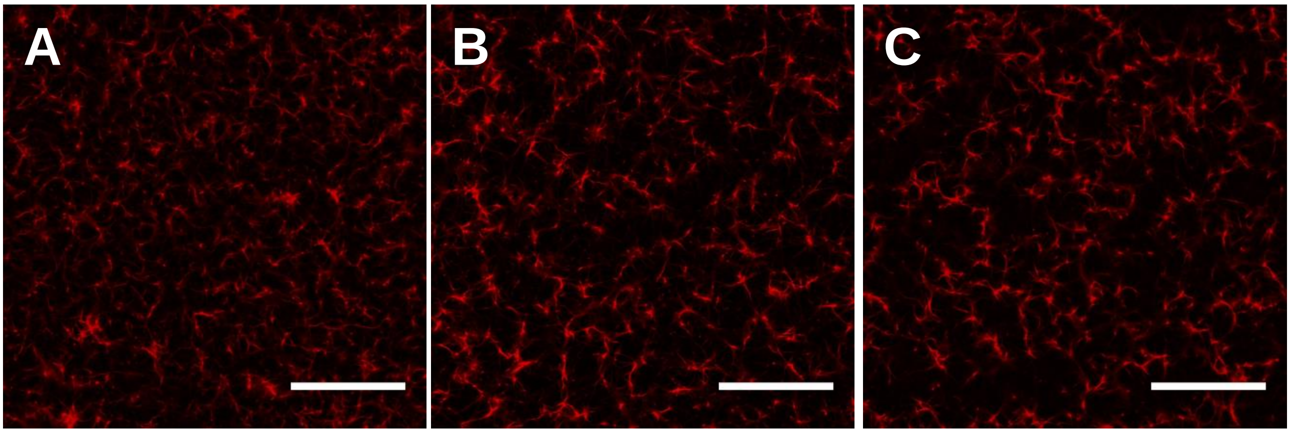

Supplementary Figure 6 | Confocal fluorescence microscopy of Alexa-594 conjugated (2 wt. %) fibrin (≈ 6 mg.mL^{-1}) gels 24 hours post formation with (≈ 0.9 µM) **A.** Native, **B.** sc_thrombin and **C.** [sc_thrombin][ox890]. Scale bars represent 50 µm.

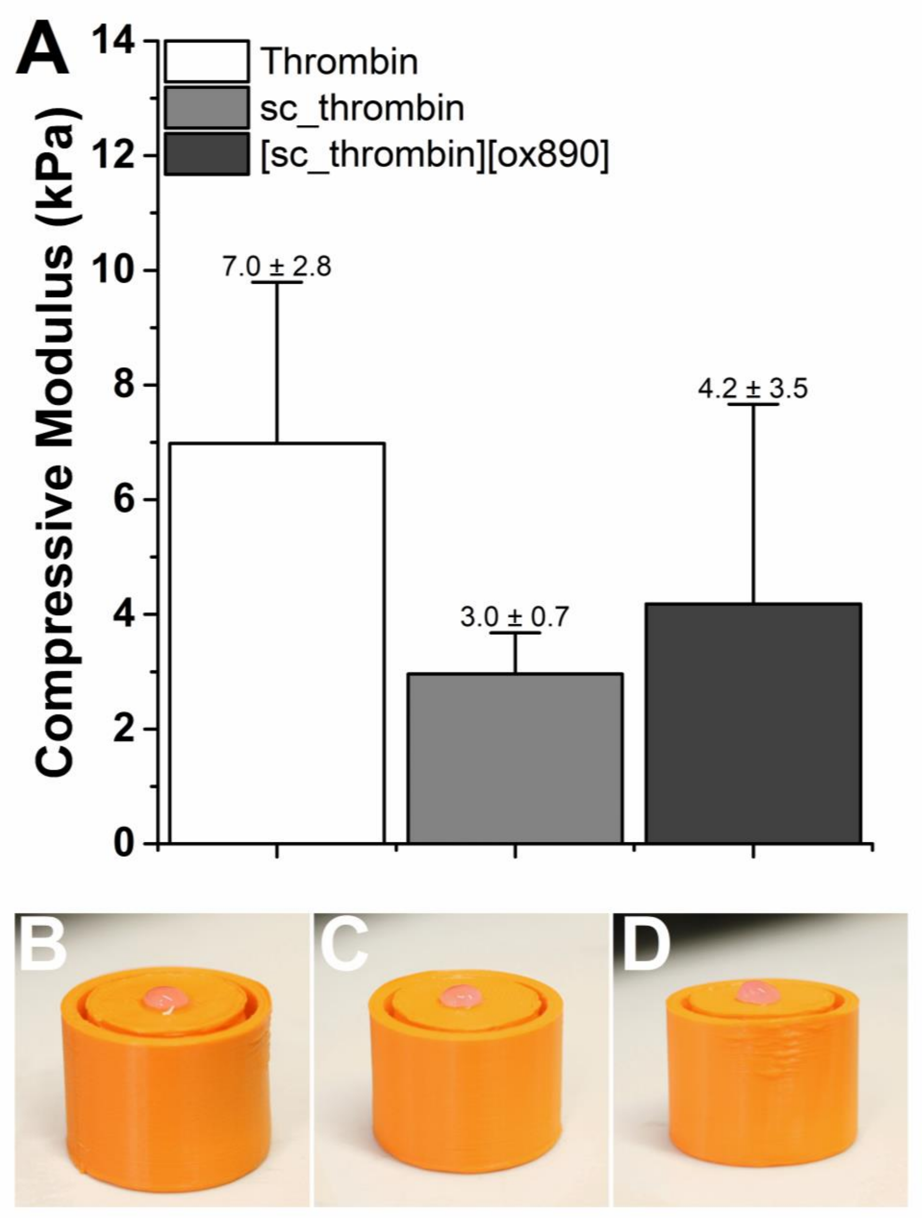

Supplementary Figure 7 | A. Compressive modulus (n=5) of 6 mg/mL^{-1} fibrin gels after 24 hours formed by 200 nM thrombin, sc_thrombin or [sc_thrombin][ox890]. Data reported as the means ± s.d. No statistically significant differences (Tukey's test). Macroscopic photographs of fibrin gels after 24 hours catalysed by 200 nM **B**. native thrombin, **C**. sc_thrombin and **D**. [sc_thrombin][ox890]. Source data are provided as a Source Data file.

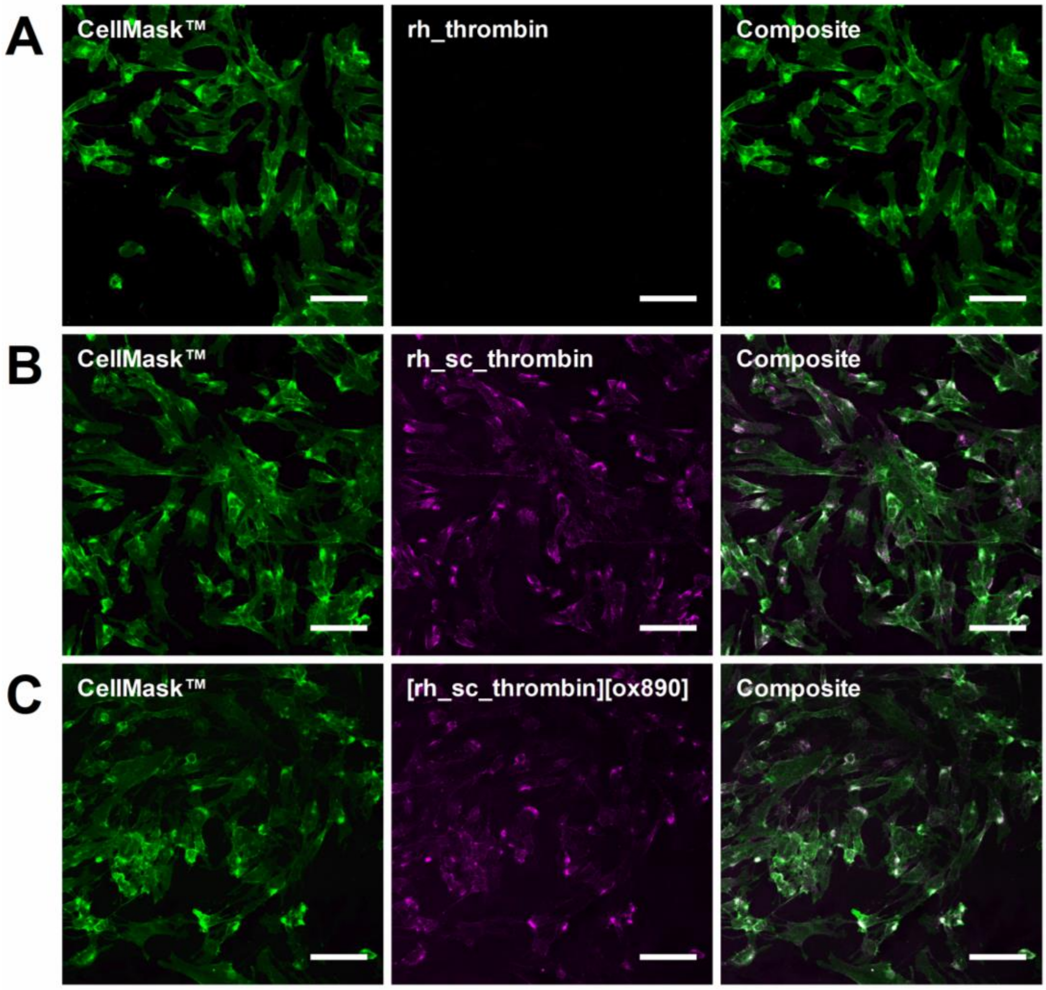

Supplementary Figure 8 | Additional evaluation of rh_thrombin, rh_sc_thrombin and [rh_sc_thrombin][ox890] hMSC plasma membrane affinity at lower magnification. Cells labelled with CellMask™ Deep Red (green) and corresponding rhodamine labelled thrombin (magenta). **A.** Native (rh_thrombin) thrombin at T= 0 hr. **B.** Cationised (rh_sc_thrombin) thrombin at T= 0 hr. **C.** Polymer surfactant conjugate ([rh_sc_thrombin][ox890]) thrombin at T= 0 hr. Scale bars represent 100 µm.

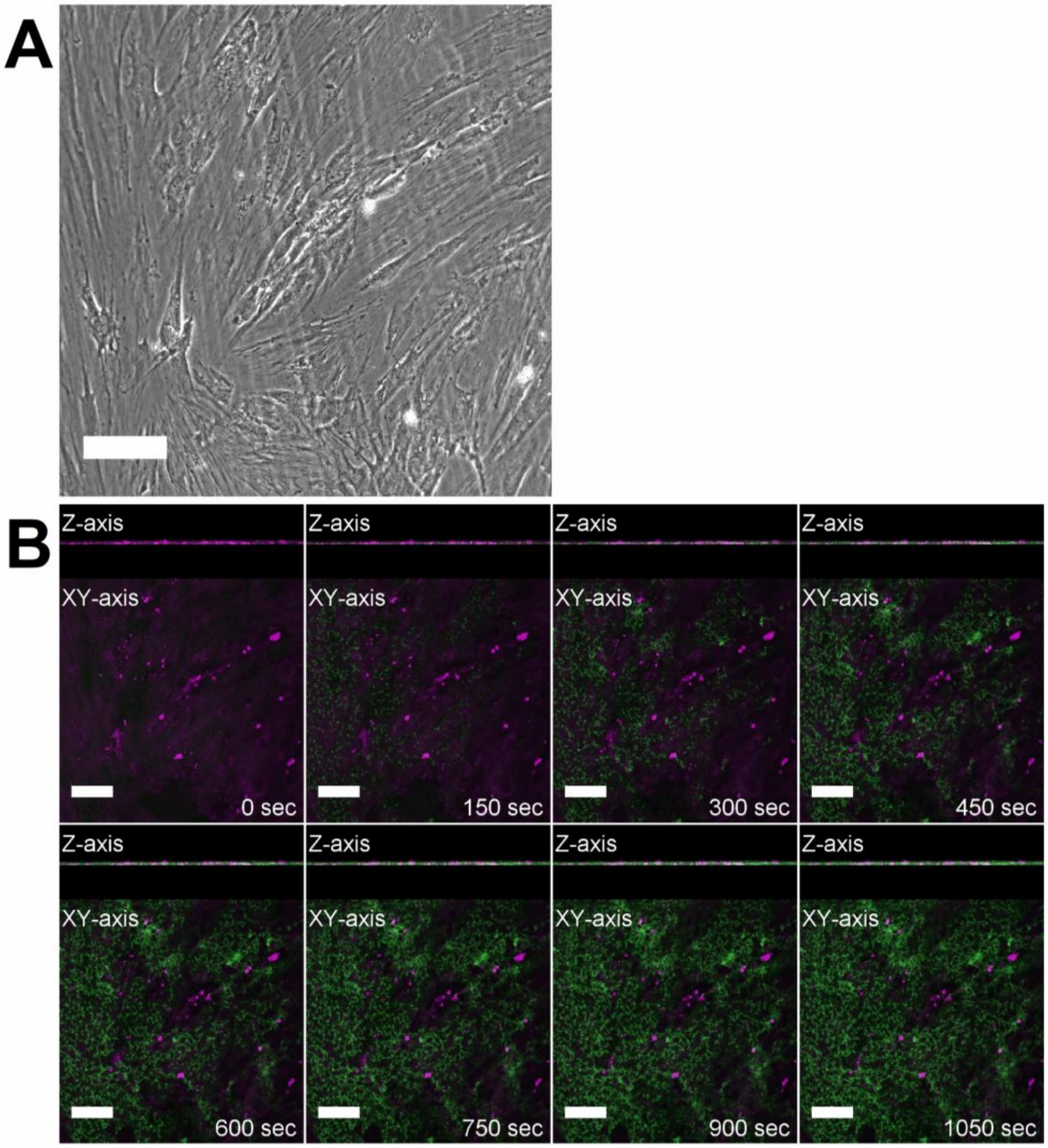

Supplementary Figure 9 | Fibrin formation from confluent [rh_sc_thrombin][ox890] coated hMSCs. **A**. Phase contrast micrograph of hMSCs at t=0 sec highlighting hMSC confluence. **B**. Time course of [rh_sc_thrombin][ox890] (magenta) labelled hMSCs supplemented with 10 mg.mL^{-1} fibrin gel comprising 1 wt.% Alexa 488 conjugated fibrinogen (green) and imaged immediately thereafter at 150 second intervals for a period of 20 minutes highlighting fibrin formation emanating from the cell surface. Scale bar represents 100 µm.

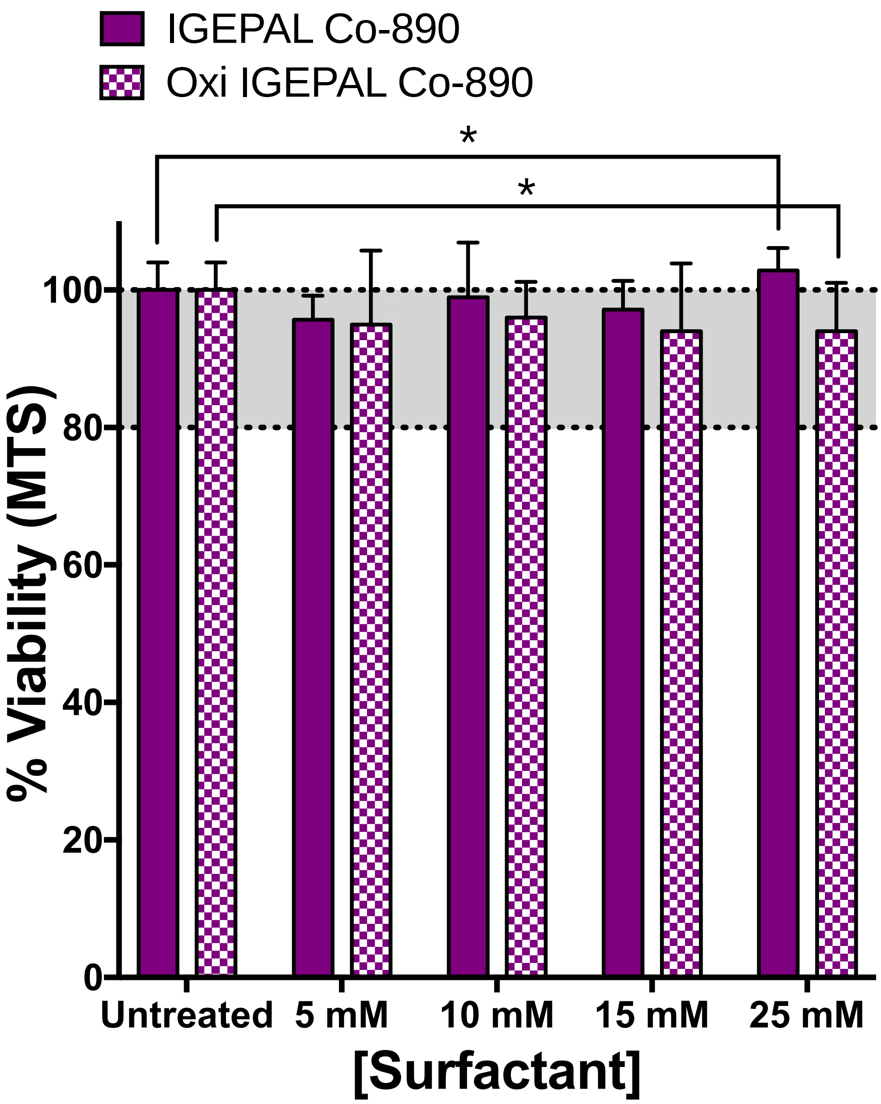

Supplementary Figure 10 | Cell viability (MTS Assay) of hMSCs exposed to either IGEPAL Co-890 or Oxidised IGEPAL Co-890 surfactant for a period of 20 minutes at 37 °C / 5 % CO_2 in 60mM HEPES solution at pH7. Data reported as means ± s.d. of 4 replicates. No significant difference (*) between untreated vs. 25 mM IGEPAL Co-890 ($p = 0.2537$) and untreated vs. Oxidised IGEPAL Co-890 ($p = 0.0958$). Two-tailed unpaired t-test. Source data are provided as a Source Data file.

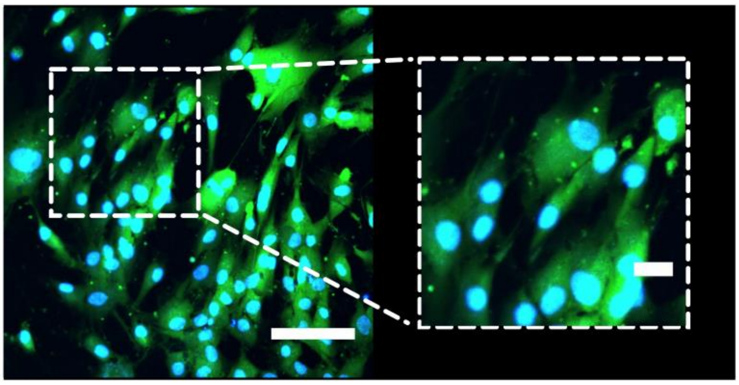

Supplementary Figure 11 | Evaluation of cellular fibrin hydrogel formation in non [sc_thrombin][ox890] modified hMSCs. Live-cell confocal fluorescence micrograph (2D) showing no spontaneous Alexa-594-labelled fibrin formation after 60 minutes in the absence of supplemented FBS. hMSCs stained with Calcein AM (green) and Hoechst 33342 (blue) with scale bar representing 100 µm. Insert represents cells at higher magnification with scale bar representing 25 µm.

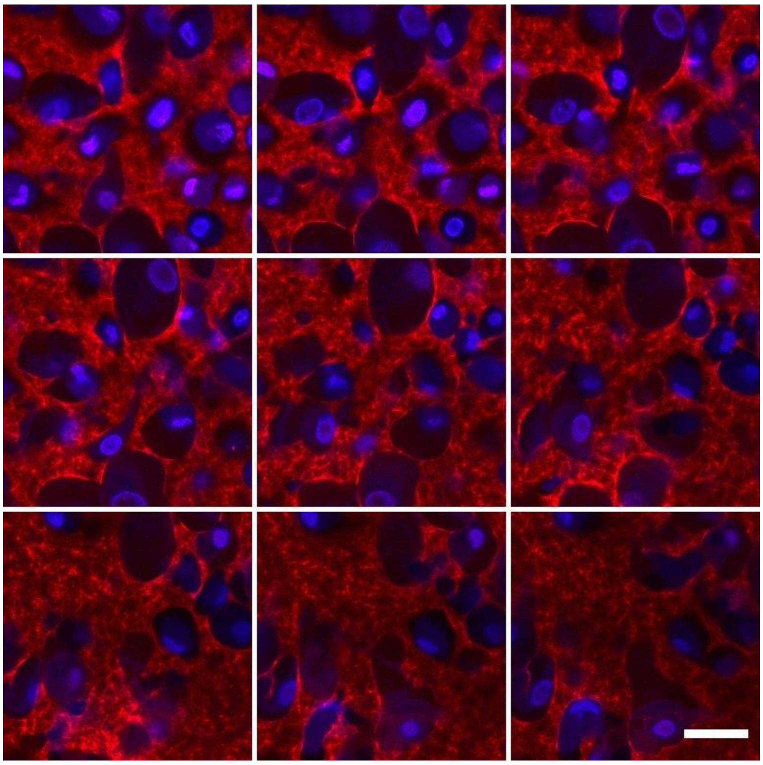

Supplementary Figure 12 | Confocal Microscopy highlighting the uniform distribution of hMSCs within an Alexa Fluor® 594 labelled fibrin gel (red) construct generated from [sc_thrombin][ox890] coated hMSCs. Cells stained with Hoechst 33342 (blue). Panels represent a Z-stack at 5 µm intervals from left to right, top to bottom. Scale bar represents 50 µm.

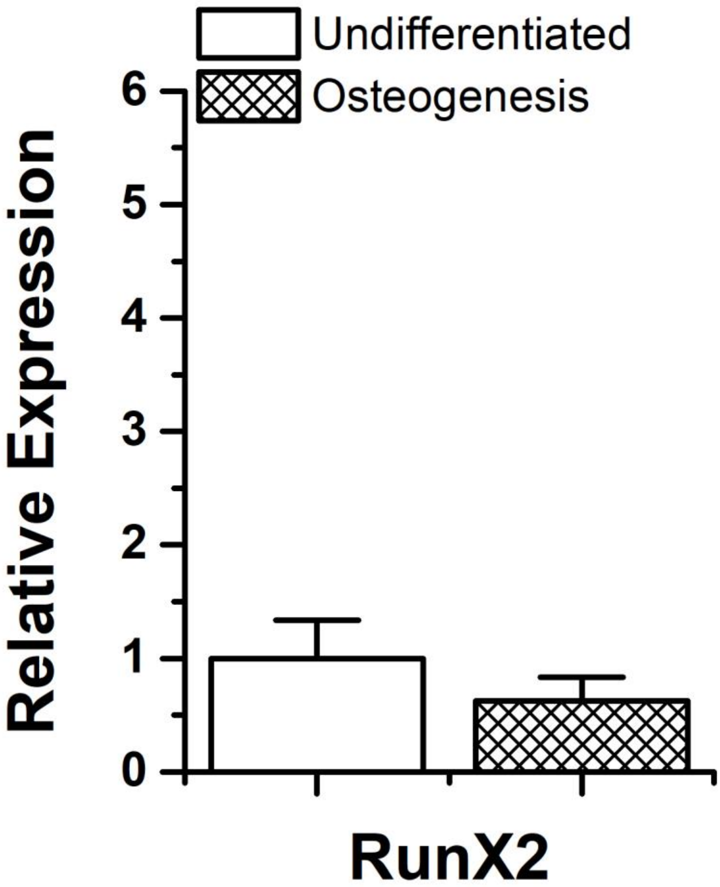

Supplementary Figure 13 | Relative expression (n≥9) of RUNX2 in [sc_thrombin][ox890] coated hMSCs within catalysed fibrin, cultured in multipotent or osteogenic medium for 7 days. Data reported as means ± s.e.m. No Significant Difference. One-tailed paired t-test; $p \leq 0.05$. Source data are provided as a Source Data file.

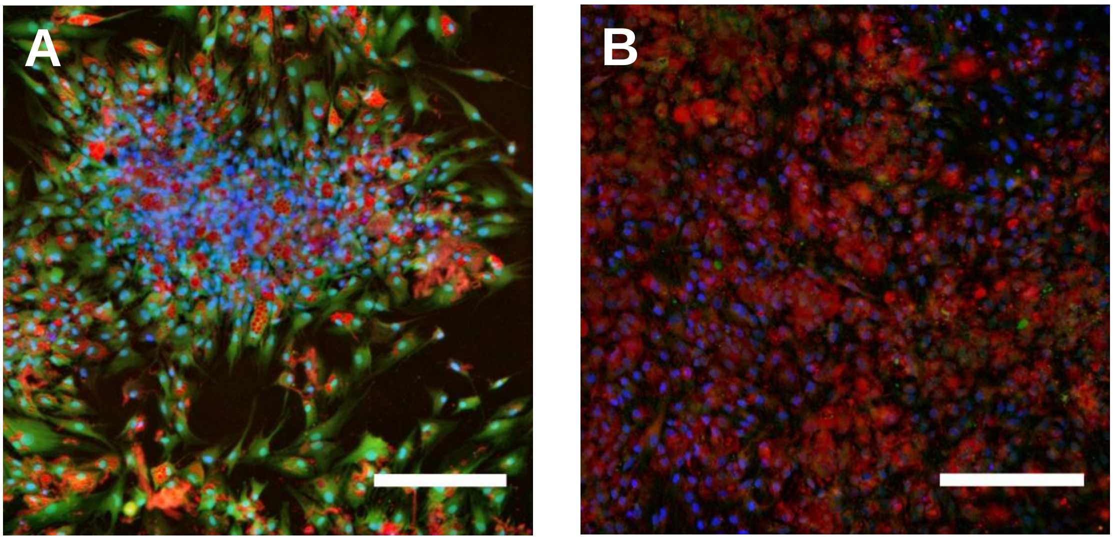

Supplementary Figure 14 | Widefield fluorescence microscopy of cells liberated (trypsin) from 3D constructs and replated as a 2D monolayer after differentiation for 21 days in adipogenic (**A.**) and osteogenic (**B.**) media. Fibrin constructs were formed initially by [sc_thrombin][ox890] labelled hMSCs prior to differentiation and liberation. All cells are stained with Calcein AM (green) and Hoechst 33342 (blue). Adipocytes additionally stained with oil red o (red) and osteocytes additionally stained with alizarin red (red). Scale bars represent 250 μm.

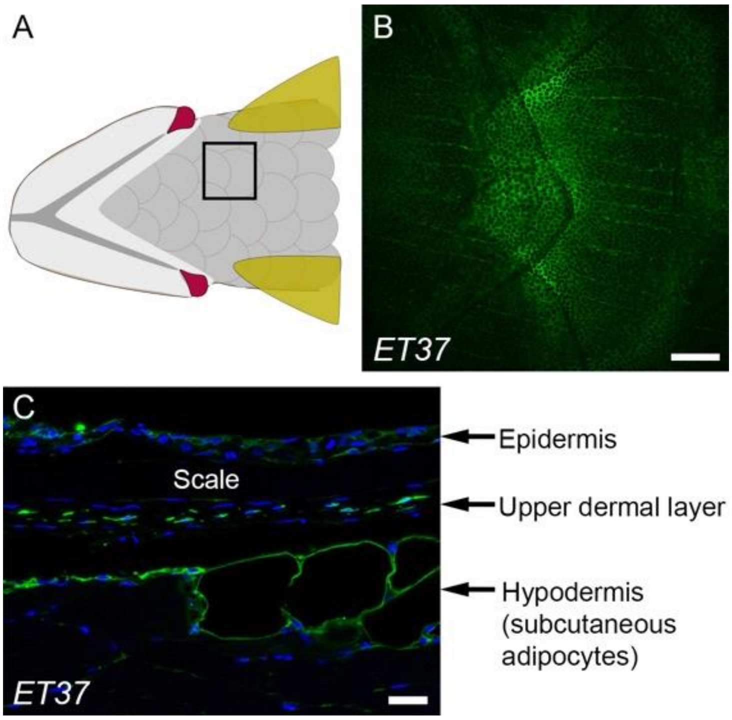

Supplementary Figure 15 | A. Schematic diagram showing the arrangement of scales on the upper ventral surface of an adult zebrafish. The boxed region indicates the approximate area shown in **B.** Confocal image of the upper ventral surface of an unwounded *ET37* transgenic zebrafish. GFP+ cells can be seen in the skin. **C.** Section through the skin of an adult *ET37* transgenic zebrafish. The majority of GFP+ cells are in the upper dermal layer and surrounding the adipocytes of the hypodermis. Scale bars: B = 200 μm; C = 20 μm.

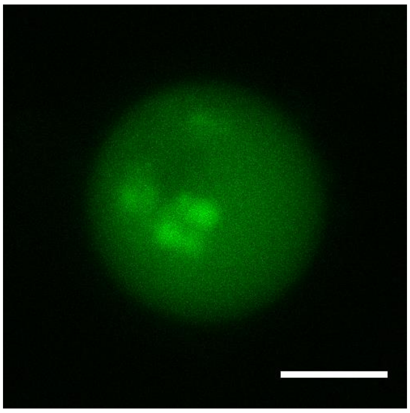

Supplementary Figure 16 | Confocal fluorescence micrograph (Z-projection) of an individual GFP expressing fibroblast from *Danio rerio* (ET37) isolated by FACs, highlighting systemic GFP expression. Scale bar represents 5 µm.

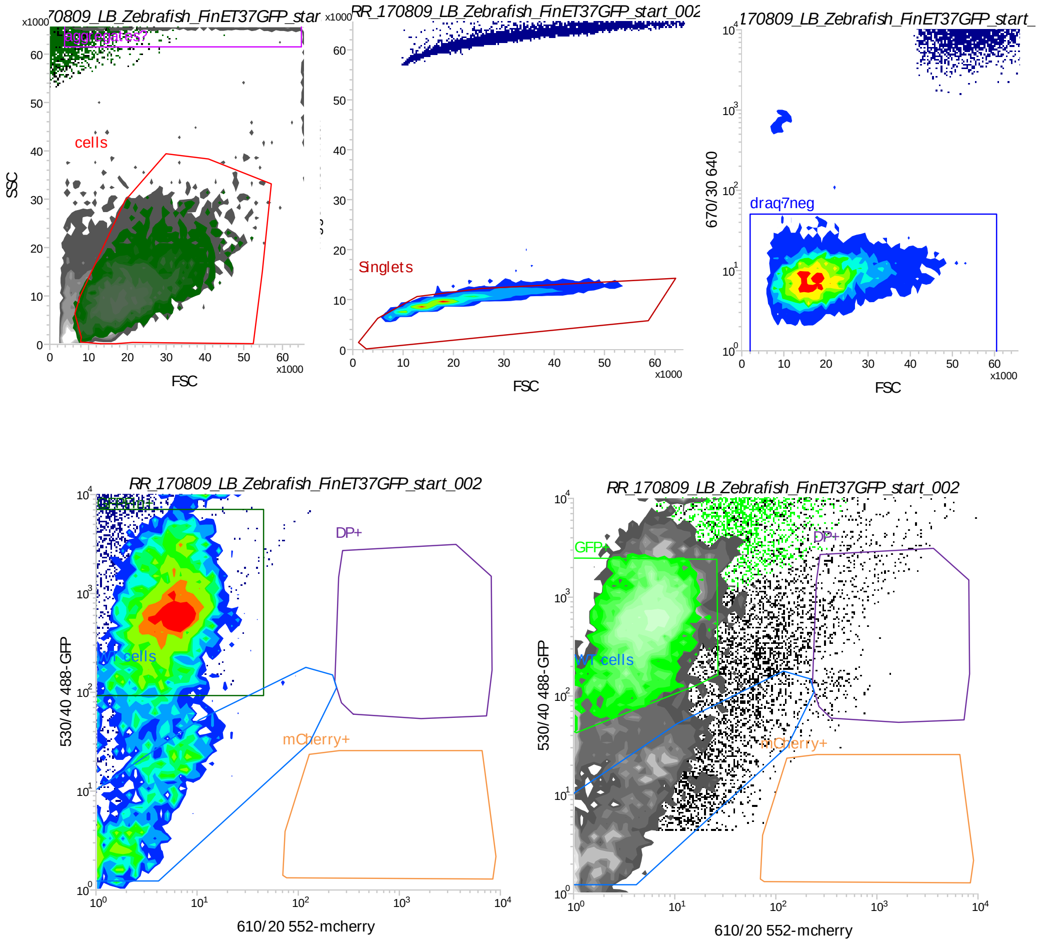

Statistics: *RR_170809_LB_Zebrafish_FinET37GFP_start_002*

Populations	Events	% Total	% Parent	FSC RCV	530/40 48... RCV	582/29 552 RCV	670/30 640 RCV	460/50 405 RCV
All Events	13,449	100.00%	####	63.22	144.86	90.29	65.31	90.04
cells	10,000	74.35%	74.35%	45.51	138.42	76.23	56.15	70.72
Singlets	8,749	65.05%	87.49%	42.06	140.89	74.91	53.14	70.42
draq7neg	8,374	62.26%	95.71%	41.25	139.78	74.91	48.60	70.45
DP+	0	0.00%	0.00%	####	####	####	####	####
mCherry+	0	0.00%	0.00%	####	####	####	####	####
GFP+	5,027	37.38%	60.03%	43.57	82.78	62.48	46.85	54.83
GFPFin+	5,547	41.24%	66.24%	40.20	87.23	61.91	46.85	52.72
WT cells	1,936	14.40%	23.12%	32.09	104.61	91.58	51.61	88.21
aggregates?	389	2.89%	2.89%	71.31	138.91	94.78	66.77	105.02
smaller scatter	2,955	21.97%	21.97%	35.25	145.16	81.35	46.14	83.03
lowscatter	2,012	14.96%	14.96%	28.37	135.13	54.45	43.88	43.41

Supplementary Figure 17 | Isolation of GFP expressing fibroblast cells from *Danio rerio* using FACS, highlighting gating criteria and isolation yields. Example plots represent heatmaps with isolation yields based on a sample of at least $1x10^3$ cells.

Supplementary Methods

Acid-Base Titration of Oxidised IGEPAL Co-890

A standard acid-base titration with 10 mL of 2.503 mM (5 $mg.mL^{-1}$) Oxidised IGEPAL Co-890 against 2.5 mM NaOH was performed with concurrent pH values measured to identify the equivalence point as the quantity of NaOH required to attain pH 7. A total of 25.025 mmol of NaOH was required to neutralise 25.03 mmol of Oxidised IGEPAL Co-890 highlighting complete and successful oxidation.

Fourier Transform Infra-Red Spectroscopy

Fourier Transform Infra-Red (FTIR) spectroscopy was performed on a Spectrum One FTIR spectrometer (Perkin Elmer Inc., MA, U.S.A). Small quantities of solid IGEPAL Co-890 and oxidized IGEPAL Co-890 were separately placed over the quartz window and the sample tightened to ensure accurate measurement. A total of 8 replicates were performed and the averages were reported. The appearance of a carbonyl stretching vibration at 1740 cm^{-1} was indicative of successful oxidation and presence of a carboxylic acid functional group

Dynamic Light Scattering

Dynamic light scattering was performed using on a Zetasizer Nano ZSP (Malvern Instruments Ltd, UK). Measurements were conducted at pH 6.5 and a temperature of 25 °C with a minimum of 300 seconds equilibration time in 20 mM HEPES. The values reported show the number (%) distribution of each individual sample.

IGEPAL Co890 & Oxidised IGEPAL Co890 Cytotoxicity

Sterile stock solutions of 5 $mg.mL^{-1}$ IGEPAL Co-890 and oxidised IGEPAL Co-890 in 60 mM HEPES pH7 were prepared and diluted in 60 mM HEPES pH7 buffer. To start, complete medium was aspirated from confluent low passage (>5) hMSCs in a 96 well plate ($1x10^4$ cells/well) and cells then rinsed with 100 µL of PBS prior to the addition of 100 µL of the appropriate solution (0-5 $mg.mL^{-1}$) was added in quadruplicate to cells and incubated for a period of 30 minutes at 37 °C. After 30 minutes cells were rinsed twice with 100 µL PBS prior to assessment of cell viability by the MTS cell viability assay.

Alexa Fluor® 594 Fibrin Gel Imaging

A 6 $mg.mL^{-1}$ solution of human fibrinogen constituting 2 wt. % Alexa Fluor® 594 conjugated human fibrinogen was made in complete medium minus FBS with single 100 µL aliquots placed within a 96-well plate. Fibrin formation was catalysed by the addition 10 µL of 0.9 µM thrombin (native, sc_thrombin or [sc_thrombin][ox890]) and samples incubated overnight at 37 °C on a lateral rotator (50 rpm) permitting mixing prior to onset of gelation. Confocal fluorescence micrographs were then collected (Excitation 594 nm; Emission 610 nm - 650 nm) the following day highlighting relative fibrin formation.

Data Analysis

Data interpretation, modelling, numerical analysis and statistical analysis were performed using Microsoft Excel for Mac 2011 (Microsoft Corporation, WA, U.S.A.) and Prism 7 for Mac OS X (Graph Pad Inc., CA, U.S.A.). Determination of sc_thrombin and [sc_thrombin][ox890] rate constants (k; min^{-1}) utilised a one-phase association model with Y=0 and Plateau =100 of the raw data (>800 data points). Statistical tests and confidence intervals detailing significant differences are as described in the figure legends of the appropriate figure.
