# Supplementary material for: Artificial cell membrane binding thrombin constructs drive in situ fibrin hydrogel formation
Source: Nat Commun. 2019 Apr 23;10:1887. doi: 10.1038/s41467-019-09763-0 (PMC6478844; doi:10.1038/s41467-019-09763-0)
Supplement: Supplementary file 2 — Description of Additional Supplementary Files [file 41467_2019_9763_MOESM2_ESM.pdf]

Description of Additional Supplementary Files

Supplementary Movie 1 | Time course (6 frames per second) from widefield fluorescence microscopy showing thrombin (magenta) labelled hMSCs in 2D over a period of 6 hours imaged at 10 minute intervals highlighting gradual internalisation of [rh_sc_thrombin][ox890] (top) and rh_sc_thrombin (bottom). Scale bar represents 100 μm.

Supplementary Movie 2 | Time course (5 frames per second) from confocal fluorescence microscopy (z-plane top; x-y plane bottom) of [rh_sc_thrombin][ox890] (magenta) labelled hMSCs supplemented with 10 mg.mL-1 fibrin gel comprising 1 wt.% Alexa 488 conjugated fibrinogen (green). Images were collected immediately after fibrinogen addition and at 30 second intervals for a period of 20 minutes, highlighting fibrin formation emanating from the cell surface. Scale bar represents 100 μm.

Supplementary Movie 3 | 3D projection (25 frames per second) of 1 mg.mL-1 fibrin gel comprising 2 wt.% Alexa 594 conjugated fibrinogen (red) and incorporating [sc_thrombin][ox890] labelled hMSCs stained with Hoechst 33342 (blue). Projection represents 111 slices each 116 μm (x) by 116 μm (y) at 0.1 μm (z) intervals collected after 60 minutes highlighting cell associated fibrin formation.

Supplementary Movie 4 | Z projection (14 frames per second) of 6 mg.mL-1 fibrin gel comprising 2 wt.% Alexa 594 conjugated fibrinogen (red) incorporating [sc_thrombin][ox890] labelled hMSCs stained with Hoechst 33342 (blue). Projection (top to bottom) represents 91 slices each 116 μm (x) by 116 μm (y) at 0.5 μm (z) intervals.

intervals.
