# Supplementary material for: Artificial cell membrane binding thrombin constructs drive in situ fibrin hydrogel formation
Source: Nat Commun. 2019 Apr 23;10:1887. doi: 10.1038/s41467-019-09763-0 (PMC6478844; doi:10.1038/s41467-019-09763-0)
Supplement: Supplementary file 7 — Reporting Summary [file 41467_2019_9763_MOESM7_ESM.pdf]

nature research

Corresponding author(s): Adam W. Perriman

Last updated by author(s): Feb 15, 2019

Reporting Summary

Nature Research wishes to improve the reproducibility of the work that we publish. This form provides structure for consistency and transparency in reporting. For further information on Nature Research policies, see Authors & Referees and the Editorial Policy Checklist.

Statistics

For all statistical analyses, confirm that the following items are present in the figure legend, table legend, main text, or Methods section.

n/a	Confirmed	
☐	☒	The exact sample size (*n*) for each experimental group/condition, given as a discrete number and unit of measurement
☐	☒	A statement on whether measurements were taken from distinct samples or whether the same sample was measured repeatedly
☐	☒	The statistical test(s) used AND whether they are one- or two-sided<br>*Only common tests should be described solely by name; describe more complex techniques in the Methods section.*
☒	☐	A description of all covariates tested
☐	☒	A description of any assumptions or corrections, such as tests of normality and adjustment for multiple comparisons
☐	☒	A full description of the statistical parameters including central tendency (e.g. means) or other basic estimates (e.g. regression coefficient) AND variation (e.g. standard deviation) or associated estimates of uncertainty (e.g. confidence intervals)
☐	☒	For null hypothesis testing, the test statistic (e.g. *F*, *t*, *r*) with confidence intervals, effect sizes, degrees of freedom and *P* value noted<br>*Give P values as exact values whenever suitable.*
☒	☐	For Bayesian analysis, information on the choice of priors and Markov chain Monte Carlo settings
☒	☐	For hierarchical and complex designs, identification of the appropriate level for tests and full reporting of outcomes
☒	☐	Estimates of effect sizes (e.g. Cohen's *d*, Pearson's *r*), indicating how they were calculated

Our web collection on statistics for biologists contains articles on many of the points above.

Software and code

Policy information about availability of computer code

Data collection	Zetasizer Software 7.12 (Malvern Instruments Ltd.)<br>Gen5 2.0 (BioTek Instruments Ltd.)<br>L2 Plus Software (The L. S. Starrett Company Ltd.)<br>StepOnePlus™ Software v2.3 (Thermo Fisher Scientific Inc.)<br>Cary WinUV 4.1 (Agilent Technologies LDA U.K. Ltd.)<br>Leica LAS-X (Leica Microsystems GmbH.)
Data analysis	Microsoft Excel for Mac 2011 (Microsoft Corporation, WA, U.S.A)<br>Prism 7 for Mac OS X (Graph Pad Inc., CA, U.S.A.)<br>OriginPro 2015 (64-bit) Sr2 (OriginLab Corp., MA, USA).

For manuscripts utilizing custom algorithms or software that are central to the research but not yet described in published literature, software must be made available to editors/reviewers. We strongly encourage code deposition in a community repository (e.g. GitHub). See the Nature Research guidelines for submitting code & software for further information.

Data

Policy information about availability of data

All manuscripts must include a data availability statement. This statement should provide the following information, where applicable:

- Accession codes, unique identifiers, or web links for publicly available datasets
- A list of figures that have associated raw data
- A description of any restrictions on data availability

All data supporting the research findings in this study are available from the corresponding author upon reasonable request. The source data underlying Figures 2b, 3d, 5a, 5b, 5c, 6c and Supplementary Figures 7a, 10 and 13 are provided as a Source Data file.

Field-specific reporting

Please select the one below that is the best fit for your research. If you are not sure, read the appropriate sections before making your selection.

☒ Life sciences ☐ Behavioural & social sciences ☐ Ecological, evolutionary & environmental sciences

For a reference copy of the document with all sections, see nature.com/documents/nr-reporting-summary-flat.pdf

Life sciences study design

All studies must disclose on these points even when the disclosure is negative.

Sample size	The use of standardized assays and techniques (cited in the manuscript) with sufficient signal to noise ratios were used where possible to ensure sufficient power was present to avoid the likelihood of type I and type II errors occurring. Consideration into sample sizing for construct formation was influenced upon the availability of donor material (i.e. hMSCs) and the retention of cell pluripotency which is typically up to passage 5 limiting cell expansion.
Data exclusions	No data exclusions
Replication	Replication where stated in the manuscript was successful
Randomization	Samples were appropriately randomized where required (e.g. compression testing)
Blinding	Blinding was utilized in the generation and analysis of all compression testing data described. Blinding was not appropriate elsewhere.

Reporting for specific materials, systems and methods

We require information from authors about some types of materials, experimental systems and methods used in many studies. Here, indicate whether each material, system or method listed is relevant to your study. If you are not sure if a list item applies to your research, read the appropriate section before selecting a response.

Materials & experimental systems

n/a	Involved in the study	
☒	☐	Antibodies
☒	☐	Eukaryotic cell lines
☒	☐	Palaeontology
☐	☒	Animals and other organisms
☐	☒	Human research participants
☒	☐	Clinical data

Methods

n/a	Involved in the study	
☒	☐	ChIP-seq
☐	☒	Flow cytometry
☒	☐	MRI-based neuroimaging

Animals and other organisms

Policy information about studies involving animals; ARRIVE guidelines recommended for reporting animal research

Laboratory animals	Wildtype and ET37 Zebrafish (Danio rerio). Age 4-18 Months. Male and Female
Wild animals	Did not involve wild animals
Field-collected samples	Did not involve animals collected from the field
Ethics oversight	University of Bristol

Note that full information on the approval of the study protocol must also be provided in the manuscript.

Human research participants

Policy information about studies involving human research participants

Population characteristics	Anonymised individuals undergoing elective orthopedic surgery
Recruitment	Individuals under going elective orthopedic surgery (e.g. hip replacement) were approached by appropriate clinical/surgical staff and provided with information detailing the use of biological material (e.g. bone marrow) that would be otherwise discarded for research purposes. This enabled the patient to make an informed decision regarding their participation. All patient information was subsequently anonymised prior to the transfer of any collected material to research staff.

Ethics oversight | North Bristol NHS Trust

Note that full information on the approval of the study protocol must also be provided in the manuscript.

Flow Cytometry

Plots

Confirm that:

☒ The axis labels state the marker and fluorochrome used (e.g. CD4-FITC).

☒ The axis scales are clearly visible. Include numbers along axes only for bottom left plot of group (a 'group' is an analysis of identical markers).

☒ All plots are contour plots with outliers or pseudocolor plots.

☒ A numerical value for number of cells or percentage (with statistics) is provided.

Methodology

Sample preparation	To obtain fibroblasts for labelling and adoptive transfer, adult ET37 fish were anaesthetised in 0.13 % MS-222 (Sigma-Aldrich Company Ltd, Cat. No. A5040) and the caudal fin resected. Pooled samples of fins from 4 fish were collected into PBS containing 10 mM HEPES, 30 mM taurine and 5.5 mM glucose and cells dissociated by the addition of 0.25 % trypsin, 12.5 M CaCl2 and 5 mg.ml-1 Collagenase II (Worthington Biochemical Corp.; Cat. No. LS004176). Dissociated cells were suspended in Zebrafish medium which comprised, Leibovitz medium (L-15) containing 0.3 mM glutamine, 0.8 mM CaCl2, 50 µg.mL-1 Streptomycin, 50 U.mL-1 Penicillin and 2 % (v/v) FBS
Instrument	InFlux high-speed Fluorescence activated cell sorter (BD Biosciences INC., San Jose, California).
Software	BD FACS™ Sortware V1.2.0.142
Cell population abundance	See Supplementary Figure 17
Gating strategy	ET37+ fibroblasts were sorted for GFP expression on a BD Biosciences InFlux high-speed Fluorescence activated cell sorter using BD FACS™ Sortware V1.2.0.142

☒ Tick this box to confirm that a figure exemplifying the gating strategy is provided in the Supplementary Information.
